# Dietary choline-derived Trimethylamine N-oxide impairs hippocampal neuronal function via PANoptosis activation

**DOI:** 10.1038/s41538-025-00599-1

**Published:** 2025-11-17

**Authors:** Yanqing Wang, Shuang Shen, Yan Sun, Shihan Zhou, Xiangrui Zhang, Zhouchenghao Song, Minjie Sun, Siyuan Jiang, Chenwei Qian, Qian Zhang, Shu Zhang, Mingqiang Wang, Boran Zhu

**Affiliations:** 1 Nanjing University of Chinese Medicine, Nanjing, 210023 Jiangsu China; 2 School of Elderly Care Services and Management, Nanjing University of Chinese Medicine, Nanjing, 210023 Jiangsu China; 3 School of Chinese Medicine, Nanjing University of Chinese Medicine, Nanjing, 210023 Jiangsu China; 4 Key Laboratory of Integrative Biomedicine for Brain Diseases, School of Chinese Medicine, Nanjing University of Chinese Medicine, Nanjing, China; 5 Jiangsu Association of Medicated Diet, Nanjing, 210023 China; 6 College of Pharmacy, Inner Mongolia Medical University, Hohhot, 010110 China; 7 Department of Surgical Oncology, Fudan University Shanghai Cancer Center, Shanghai, 200032 China

**Keywords:** Cell death in the nervous system, Risk factors, Apoptosis, Necroptosis

## Abstract

The gut microbial metabolite Trimethylamine N-oxide (TMAO) is increasingly implicated in the functioning and pathology of the central nervous system. Here, we demonstrate that chronic systemic TMAO exposure in mice induces significant cognitive impairment, as shown by significant deficits across multiple metrics in a battery of behavioral tests, including the novel object recognition, Y-maze, and Morris water maze (*p* < 0.05). This behavioral deficit was associated with severe hippocampal neurodegeneration, including a 20.5% loss of pyramidal neurons in the CA1 subregion, and marked mitochondrial damage. Mechanistically, TMAO-induced neurotoxicity was driven by PANoptosis, a coordinated inflammatory cell death pathway. We observed robust activation of the sensor ZBP1 and downstream executioner proteins, including cleaved Caspase-1/-3/-8, phosphorylated MLKL, and subsequent gasdermin D-mediated membrane pore formation. Crucially, pharmacological co-inhibition of RIPK3 (GSK-872) and caspases (Emricasan) significantly rescued neuronal viability, confirming PANoptosis as the core pathogenic pathway. These findings establish a novel mechanistic link between a gut-derived metabolite and cognitive decline, identifying TMAO possesses neurotoxicity that drives neurodegeneration via PANoptotic cell death. Our work suggests that strategies targeting systemic TMAO levels may hold therapeutic potential for neurodegenerative disorders.

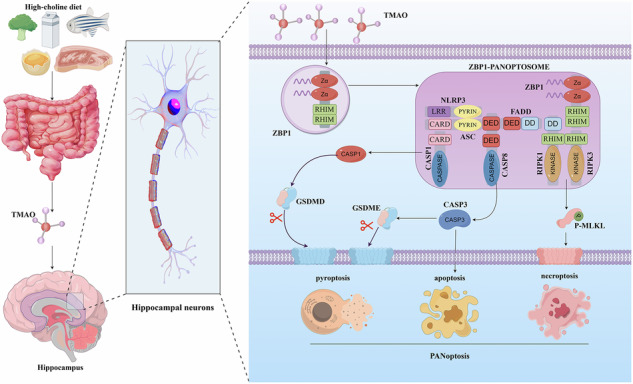

## Introduction

Mild Cognitive Impairment (MCI) is recognized as an intermediary phase between normal cognitive aging and dementia, typically marked by deficits across multiple cognitive domains^1^. Unlike the progressive and largely irreversible nature of neurodegenerative diseases like Alzheimer’s (AD) and Parkinson’s (PD), MCI often progresses more gradually, thereby presenting a critical opportunity for interventions aimed at delaying or preventing the onset of dementia^2^. In light of the absence of effective treatments to halt neurodegeneration, early-stage intervention strategies, particularly those focusing on dietary factors, have garnered increasing interest. Consequently, the potential influence of the Western diet, characterized by high-fat and high-choline consumption, on cognitive impairment is receiving growing scholarly attention. Choline is vital for producing acetylcholine, a neurotransmitter integral to memory and learning^3^. However, Excessive choline intake has been linked to metabolic syndrome, cardiovascular disease, neurological disorders^4^, and certain cancers^5^. Choline is essential for human health, yet dietary guidelines remain inconsistent, and its safe upper intake level is unclear^6^. Growing evidence suggests that excessive choline intake may elevate circulating levels of trimethylamine N-oxide (TMAO)—a gut microbiota-derived metabolite of choline, betaine, and L-carnitine^7–9^—which has been implicated in cardiovascular diseases such as atherosclerosis, myocardial infarction, and heart failure^10–14^, and is increasingly recognized as a predictor of adverse cardiovascular outcomes and mortality^15,16^. In recent years, research on TMAO has increasingly focused on its implications for neuropathy and mental illness^17^. TMAO is recognized as a risk factor for cognitive impairment^18^. Studies suggest that TMAO can cross the blood-brain barrier (BBB) and influence the central nervous system, potentially contributing to the pathogenesis of neurodegenerative diseases such as AD and PD^19^. The mechanisms may involve inflammatory pathway activation and oxidative stress induction, potentially causing neuronal damage and cognitive decline. Thus, TMAO, a microbial metabolite of choline, is identified as a potential neurotoxin. However, the mechanism linking dietary TMAO exposure to hippocampal neurodegeneration has not yet been fully explored.

PANoptosis is a novel concept in cell death pathways, combining the mechanisms of pyroptosis, apoptosis, and necroptosis into a distinct form of programmed cell death^20^. This integrated death modality serves as a critical host defense strategy, enabling the elimination of infected or damaged cells even in the presence of microbial inhibitors targeting individual death pathways^21^. By circumventing such evasion mechanisms, PANoptosis not only amplifies the inflammatory response but also contributes to the pathophysiology of diverse infectious, autoimmune, and neurodegenerative disorders. Pyroptosis, typically associated with inflammatory responses, involves the formation of pores in the cell membrane by gasdermin proteins, leading to cell lysis and the release of pro-inflammatory cytokines^22–24^. Apoptosis, on the other hand, is a more controlled form of cell death that involves the activation of caspases, leading to cell shrinkage and DNA fragmentation without eliciting an inflammatory response^25–27^. Necroptosis is a caspase-independent form of cell death that is mediated by receptor-interacting protein kinases (RIPK1 and RIPK3) and the pseudokinase MLKL, resulting in cell membrane rupture and inflammation^28,29^. Within the PANoptosis process, key proteins associated with pyroptosis, apoptosis, and necroptosis converge to form a multi-protein complex known as the PANoptosome^30^. To date, researchers have identified four primary PANoptosome scaffold prototypes: the PANoptosome associated with Z-DNA binding protein 1 (ZBP1), which emerges during influenza A virus (IAV) infection^31^; the PANoptosome mediated by receptor-interacting protein kinase 1 (RIPK1), which appears in response to herpes simplex virus 1 (HSV-1) or Neisseria infection^32^; the PANoptosome assembled by Absent in Melanoma 2 (AIM2), which is activated by sensing foreign or endogenous DNA in the cytoplasm^33^; and the PANoptosome that utilizes the NLR family member NLRP12 as a scaffold to respond to specific pathogen signals^34^. Understanding the molecular mechanisms underlying PANoptosis and its regulation is essential for developing novel therapeutic strategies. Modulating the PANoptosome components or the interaction between pyroptosis, apoptosis, and necroptosis may offer novel therapeutic strategies for diseases marked by abnormal cell death and inflammation. As research in this area continues to evolve, PANoptosis holds promise as a potential target for therapeutic intervention in a wide range of pathological conditions^35–37^.

PANoptosis, has emerged as a critical mechanism in the pathogenesis of neurodegenerative disorders^38^. In the context of Alzheimer’s disease (AD), PANoptosis is increasingly recognized as a key driver of neuroinflammation and neuronal loss. Studies have established that pathological protein aggregates, such as amyloid-beta (Aβ), can activate core components of the PANoptosome, like the NLRP3 inflammasome, in microglia^39^. Rajesh et al. highlighted that key molecules driving PANoptosis (such as AIM2, CASP8, and RIPK3) are central to the neuroinflammation seen in Alzheimer’s disease (AD)^40^. Building on this, Meng et al. proposed therapeutic strategies that target how amyloid-beta oligomers (AβOs) trigger PANoptosis through mitochondrial damage. Their suggestions include inhibiting the mitochondrial permeability transition pore (mPTP) to prevent the release of harmful factors like mitochondrial DNA (mtDNA) and reactive oxygen species (ROS)^41^. Another study proposed that the AIM2-PANoptosome contributes to AD pathogenesis, since eliminating AIM2 in a 5xFAD mouse model reduced both Aβ plaques and microglial activation^42^. On the clinical front, Zhang et al. developed a predictive tool called a PANscore. By using LASSO regression analysis on PANoptosis-related genes, this model can effectively forecast a patient’s prognosis, helping doctors create personalized treatment plans for AD^43^. Understanding these alternative triggers is essential, as they may represent a distinct and previously overlooked axis of the gut-brain connection in neurodegeneration.

In our previous work, we demonstrated that TMAO can induce significant neurotoxic effects^44^. Building upon this foundational finding, a critical remaining question is the precise molecular mechanism driving this neuronal damage. Given that TMAO is well-documented to cross the blood-brain barrier, making it a plausible pathogenic agent in the central nervous system^45,46^, the present study was designed to dissect the cell death pathways triggered by this metabolite, using our established concentration. This research explored how TMAO influences PANoptosis in hippocampal neurons and its impact on cognitive deficits in mice. Central to this investigation is the hypothesis that TMAO intervention is likely to exacerbate the PANoptosis of hippocampal neurons in mice with cognitive impairment, thereby further impairing their cognitive function.

## Results

### TMAO intervention does not cause movement disorder or anxiety in mice

**Fig. 1:**
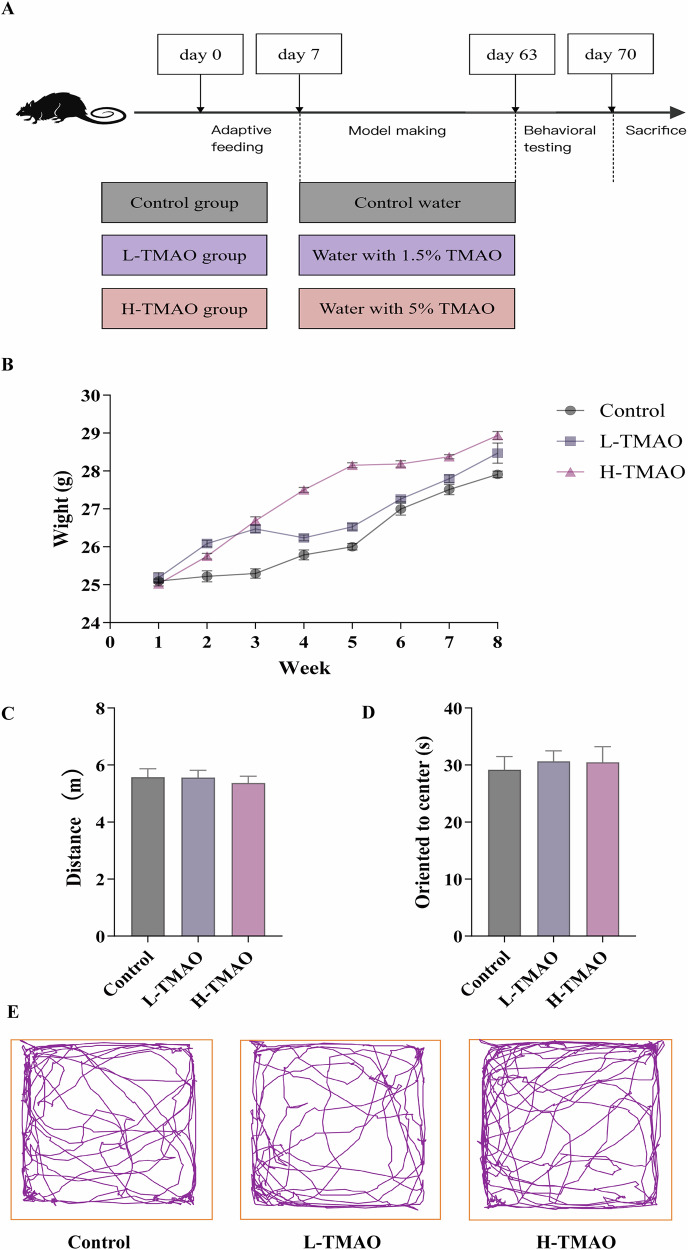
Experimental design and behavioral assessment in TMAO-treated mice. **A** Schematic of the experimental timeline. **B** Body weight was monitored weekly for 8 weeks. **C** Total distance traveled in the open field test. **D** Time spent by mice moving towards the center in the open field test. **E** Representative tracking paths during the open field test for each treatment group. Data represented by Mean with SEM, *n* = 8 per group.

The experimental design is illustrated in Fig. 1A. Mice were first subjected to a 7-day adaptive feeding period, followed by model induction through administration of either standard water (control group), water containing 1.5% TMAO (L-TMAO group), or water containing 5% TMAO (H-TMAO group) from day 7 to day 63. Behavioral assessments were conducted between days 63 and 70, after which all animals were sacrificed.

Body weight was monitored weekly throughout the experimental period (Fig. 1B). All groups exhibited a gradual increase in body weight over the 8-week period. Although mice in the L-TMAO and H-TMAO groups appeared to gain slightly more weight than controls at certain time points, no statistically significant differences were observed, suggesting that TMAO administration had no marked impact on overall weight gain.

Recent research found that infusing TMAO into the hippocampus during adulthood changed social behaviors^47^. It is unclear whether TMAO absorbed by the digestive system can cause motor impairment or anxious behavior in mice. To evaluate locomotor activity and anxiety-like behaviors, open-field testing was performed at the end of the treatment period. Total distance traveled in the arena was comparable among the control, L-TMAO, and H-TMAO groups (Fig. 1C), indicating that TMAO did not impair spontaneous locomotion. Furthermore, the time oriented toward the center of the arena, a measure often associated with anxiety-related behavior, showed no significant differences among the groups (Fig. 1D). Representative movement trajectories recorded during the open-field test further support these findings (Fig. 1E). Mice from all three groups exhibited similar patterns of exploration, with no obvious alterations in spatial behavior.

Taken together, these results suggest that chronic TMAO administration, at both low and high doses, does not significantly affect body weight, locomotor activity, or anxiety-related behavior in mice under the tested conditions.

### TMAO intervention impairs spatial long-term working memory in mice

**Fig. 2:**
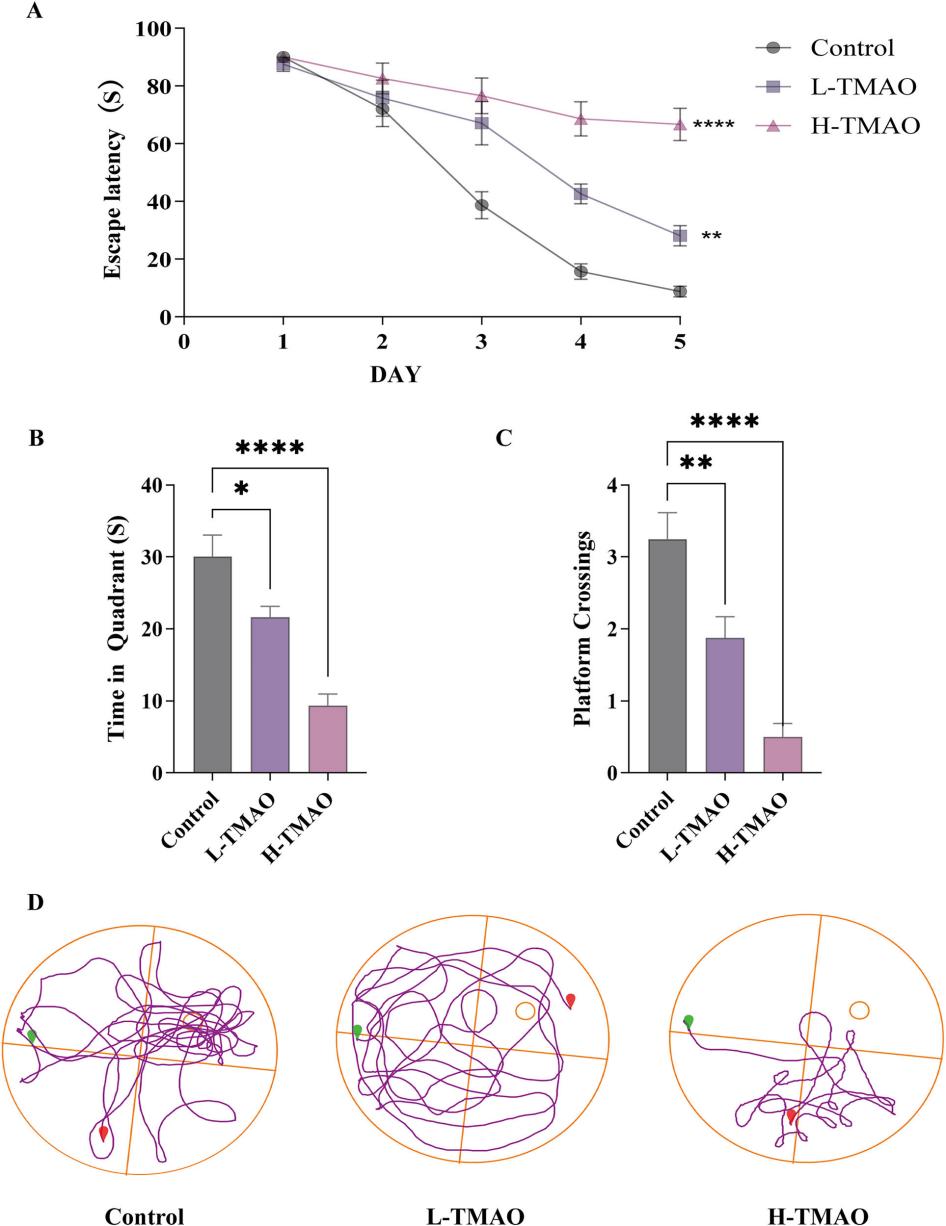
Spatial learning and memory performance in the Morris water maze following TMAO treatment. **A** Escape latency during the 5-day training phase of the Morris water maze test. **B** Time spent in the target quadrant during the probe trial. **C** Number of platform crossings during the probe trial. **D** Representative swimming trajectories during the probe trial for control, L-TMAO, and H-TMAO groups. Green dot indicates the starting position; red dot indicates the former platform location. Data are presented as mean ± SEM. *p < 0.05, **p < 0.01, ***p < 0.001, *n* = 8 per group.

To assess the impact of TMAO on cognitive function, the Morris water maze test was conducted to evaluate spatial learning and memory performance in control, L-TMAO, and H-TMAO treated mice. During the 5-day training phase (Fig. 2A), mice in the control group exhibited a progressive reduction in escape latency, indicating effective learning. In contrast, both L-TMAO and H-TMAO groups showed significantly prolonged escape latencies across days, particularly on days 4 and 5, demonstrating impaired spatial learning. The H-TMAO group showed the greatest deficits compared to control. In the probe trial conducted on day 6, the time spent in the target quadrant (Fig. 2B) and the number of platform crossings (Fig. 2C) were significantly reduced in the L-TMAO and H-TMAO groups relative to control. Notably, H-TMAO treatment resulted in the most pronounced reduction in both metrics, indicating substantial memory impairment. Representative swimming paths (Fig. 2D) further supported these findings. Control mice exhibited focused search behavior around the former platform location, while TMAO-treated mice, especially in the H-TMAO group, demonstrated disorganized search patterns and reduced exploration of the target quadrant.

### TMAO intervention impairs spatial short-term working memory in mice

To assess the impact of TMAO on cognitive performance, mice were subjected to Y-maze and novel object recognition (NOR) behavioral assays.

**Fig. 3:**
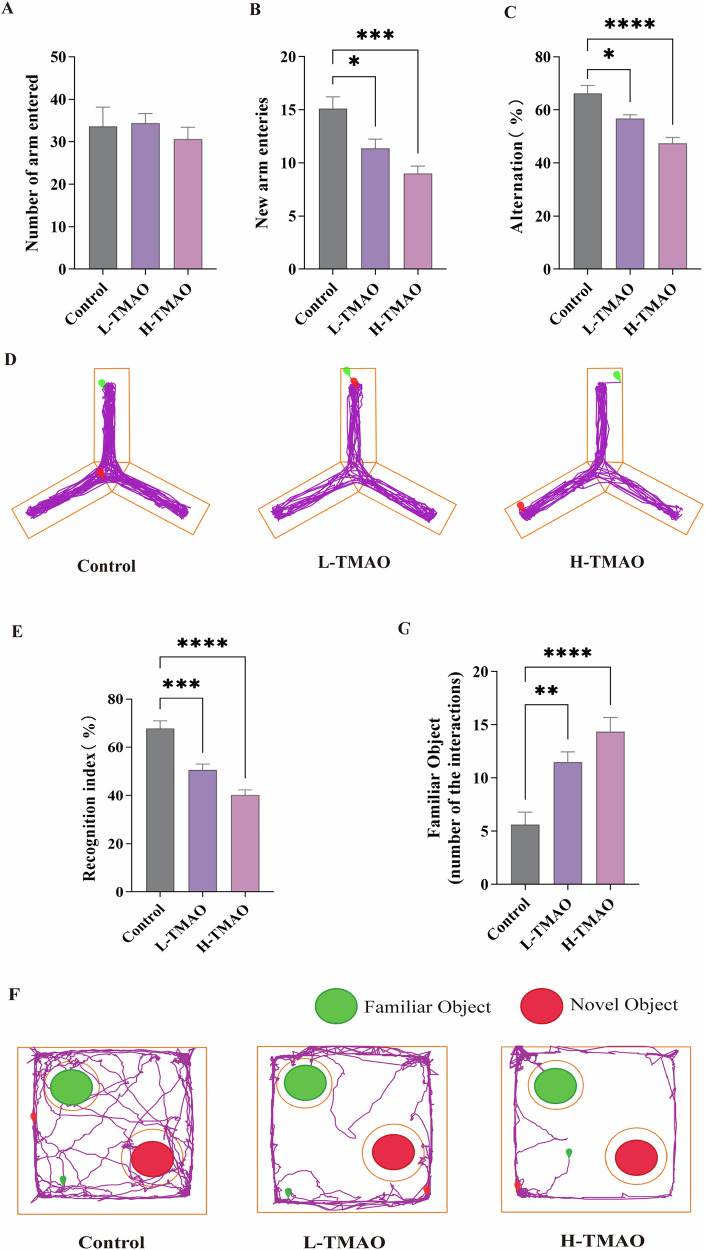
Behavioral assessments of working memory and recognition memory following TMAO treatment. **A** Total number of arm entries. **B** Number of new arm entries. **C** Percentage of spontaneous alternation. **D** Representative movement traces in the Y-maze from each group. **E** Recognition index. **F** Representative exploration tracks during the NOR test. Green circles represent familiar objects; red circles represent novel objects. **G** Number of interactions with the familiar object. Data are presented as mean ± SEM. *p < 0.05, **p < 0.01, ***p < 0.001, ****p < 0.0001. *n* = 8 per group.

In the Y-maze test, total arm entries did not differ significantly among the control, L-TMAO, and H-TMAO groups, indicating comparable locomotor activity across groups (Fig. 3A). However, the number of new arm entries was significantly reduced in both L-TMAO and H-TMAO-treated mice compared to controls, suggesting impaired exploratory behavior (Fig. 3B). Furthermore, the percentage of spontaneous alternation, an indicator of working memory, was markedly decreased in the L-TMAO and H-TMAO groups relative to controls, with the H-TMAO group displaying the most profound impairment (Fig. 3C). Representative movement traces in the Y-maze confirmed reduced exploratory behavior in TMAO-treated mice (Fig. 3D).

In the novel object recognition test, recognition index was significantly lower in both L-TMAO and H-TMAO groups compared to controls, indicating impaired recognition memory (Fig. 3E). Heatmap-like movement tracks further demonstrated reduced preference for the novel object in TMAO-treated animals (Fig. 3F). Quantification of object interaction showed significantly more interactions with the familiar object in TMAO-treated mice, suggesting overall diminished exploratory motivation and recognition discrimination (Fig. 3G).

Collectively, these data suggest that TMAO impairs both working memory and recognition memory in a dose-dependent manner, without significantly affecting general locomotor activity.

### TMAO induces neuronal loss and mitochondrial damage in the hippocampus

**Fig. 4:**
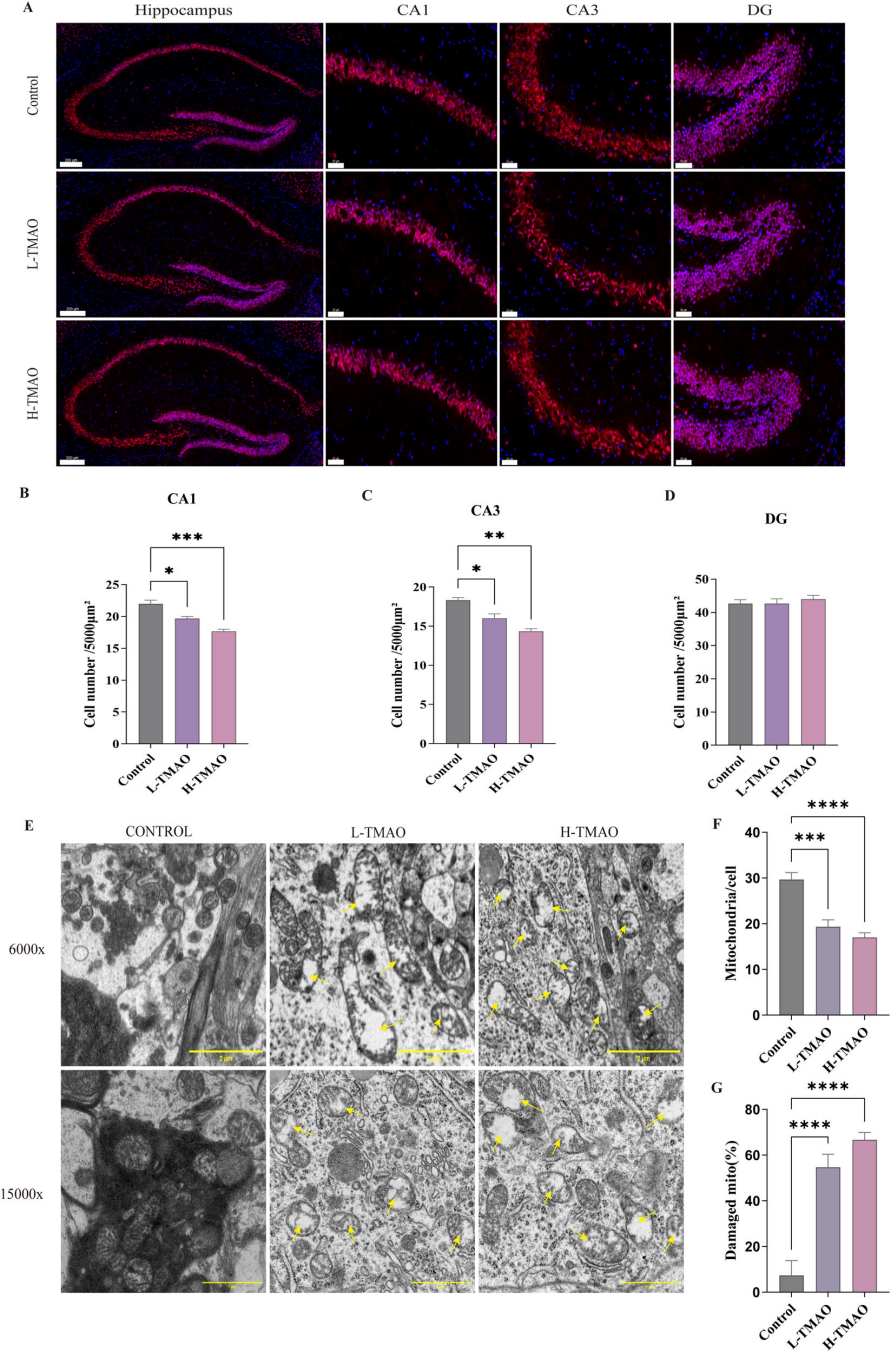
TMAO intervention leads to neuronal loss and Neuronal mitochondria damage. **A** Representative sections of the Immunofluorescent staining of the various hippocampal areas CA1, CA3, and dentate gyrus (DG) in Control, LTMAO, and HTMAO groups; **B**–**D** group data of the counted Neurons stained with immunofluorescence per 5000μm² in the hippocampal areas: CA1 (**B**), CA3 (**C**), and DG (**D**); **E** representative the ultrastructure of hippocampal neurons in the HIP of three groups on TEM (x6000 and x15,000). Yellow arrow: the vacuolate degeneration of massive mitochondria. **F** Quantification of mitochondrial numbers; Scale bars: 1,2 μm. **G** Quantification of mitochondrial damage percentage. Data analyzed by one-way ANOVA, represented by Mean with SEM, *P < 0.05, **P < 0.01, ***P < 0.001, ****P < 0.0001, *n* = 3 per group.

To evaluate the neurotoxic effects of TMAO in the hippocampus, we performed immunofluorescence staining and transmission electron microscopy (TEM) analysis in different hippocampal subregions. As shown in Fig. 4A, NeuN immunostaining revealed a progressive reduction in neuronal density in the CA1 and CA3 regions following TMAO exposure, with the most pronounced loss observed in the high-dose group (H-TMAO). Quantitative analysis demonstrated a significant decrease in the number of NeuN-positive cells in the CA1 (Fig. 4B) and CA3 (Fig. 4C) regions in both low-dose (L-TMAO) and H-TMAO groups compared to the control. In contrast, no significant differences were observed in the dentate gyrus (DG) region among the groups (Fig. 4D).

To further observe the ultrastructure of hippocampal neurons in response to TMAO intervention, we used transmission electron microscopy to observe mouse hippocampal neurons. Ultrastructural analysis by TEM (Fig. 4E) revealed a marked increase in mitochondrial abnormalities, including swelling, cristae disruption, and vacuolization, in both TMAO-treated groups. Mitochondrial number per cell was significantly reduced in the L-TMAO and H-TMAO groups (Fig. 4F), while the proportion of damaged mitochondria was significantly elevated (Fig. 4G), indicating severe mitochondrial dysfunction.

### Mouse hippocampal tissue expression of PANoptosis-related proteins is elevated

**Fig. 5:**
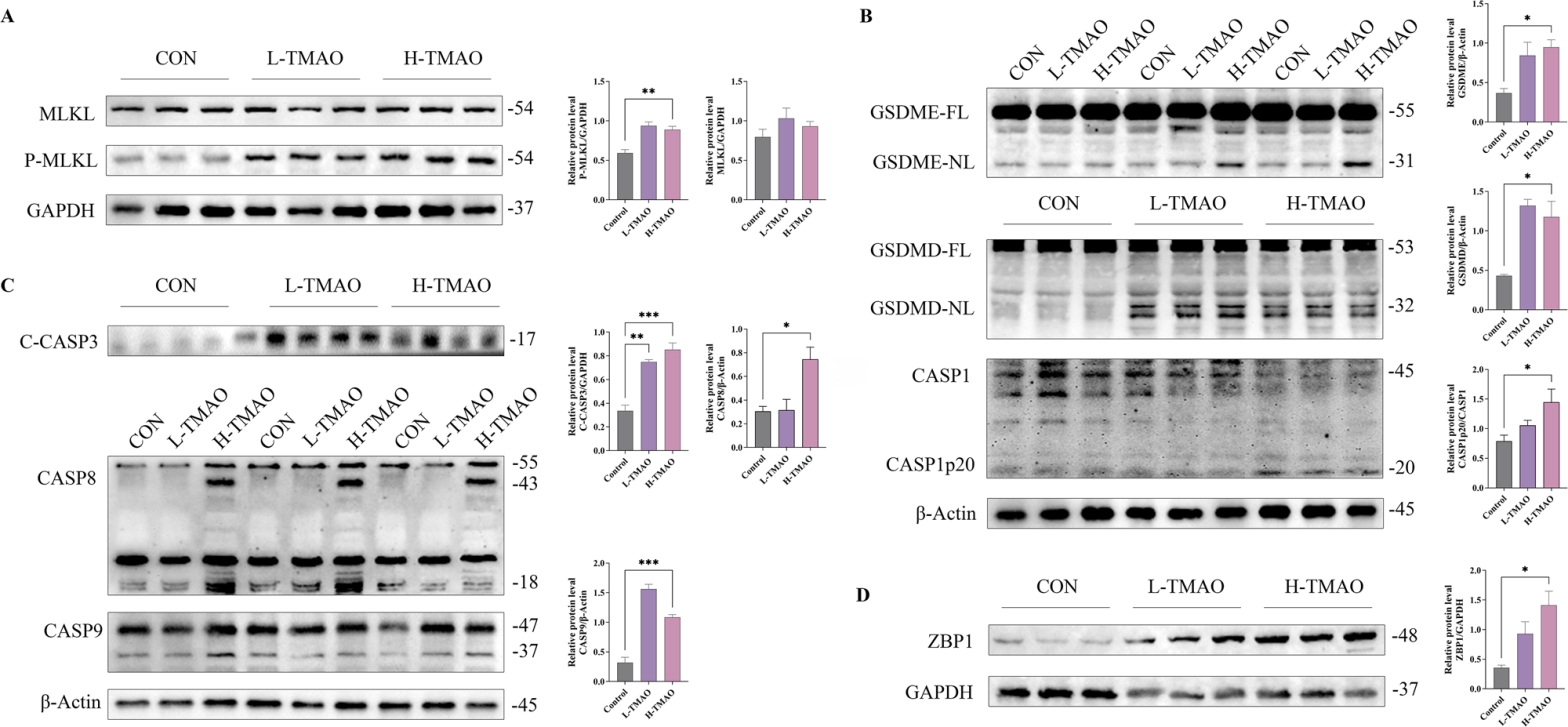
Protein expression of key genes for PANoptosis. **A** Expression of necroptosis-related proteins (MLKL, P-MLKL) in Control, L-TMAO, and H-TMAO groups. **B** Expression of pyroptosis-related proteins (CASP1, GSDMD, GSDME) in Control, L-TMAO, and H-TMAO groups. **C** Expression of apoptosis-related proteins (CASP3, CASP8, CASP9) in Control, L-TMAO, and H-TMAO groups. **D** Protein expression of Zbp1 in Control, L-TMAO, and H-TMAO groups. Standardization strategies use GAPDH/β-actin. Data analyzed by one-way ANOVA, *n* = 3, represented by Mean with SEM, *P < 0.05, **P < 0.01, ***P < 0.001, *n* = 3 per group.

To examine the impact of TMAO on various forms of programmed cell death, we assessed the expression levels of proteins associated with necroptosis (MLKL, P-MLKL), pyroptosis (CASP1, GSDMD, GSDME), and apoptosis (CASP3, CASP8, CASP9) in hippocampal tissue. As illustrated in Fig. 5A, P-MLKL was significantly elevated in the H-TMAO group compared to the control, while total MLKL levels remained consistent across all groups. Fig. 5B reveals that TMAO treatment facilitated the cleavage of GSDME and GSDMD, with the cleaved forms (GSDME-NL and GSDMD-NL) substantially upregulated in the H-TMAO group. Furthermore, the cleaved form of caspase-1 (CASP1p20) was significantly increased following TMAO treatment, indicating activation of the caspase-1-dependent pyroptotic pathway. As depicted in Fig. 5C, levels of cleaved caspase-3 (C-CASP3) were significantly elevated in the H-TMAO group, accompanied by upregulation of caspase-8 (CASP8) and caspase-9 (CASP9), suggesting that TMAO also activates apoptotic signaling pathways. Figure 5D demonstrates that ZBP1 expression was significantly increased following TMAO treatment, with the highest expression observed in the H-TMAO group.

### TMAO induces dose-dependent cytotoxicity and apoptosis

**Fig. 6:**
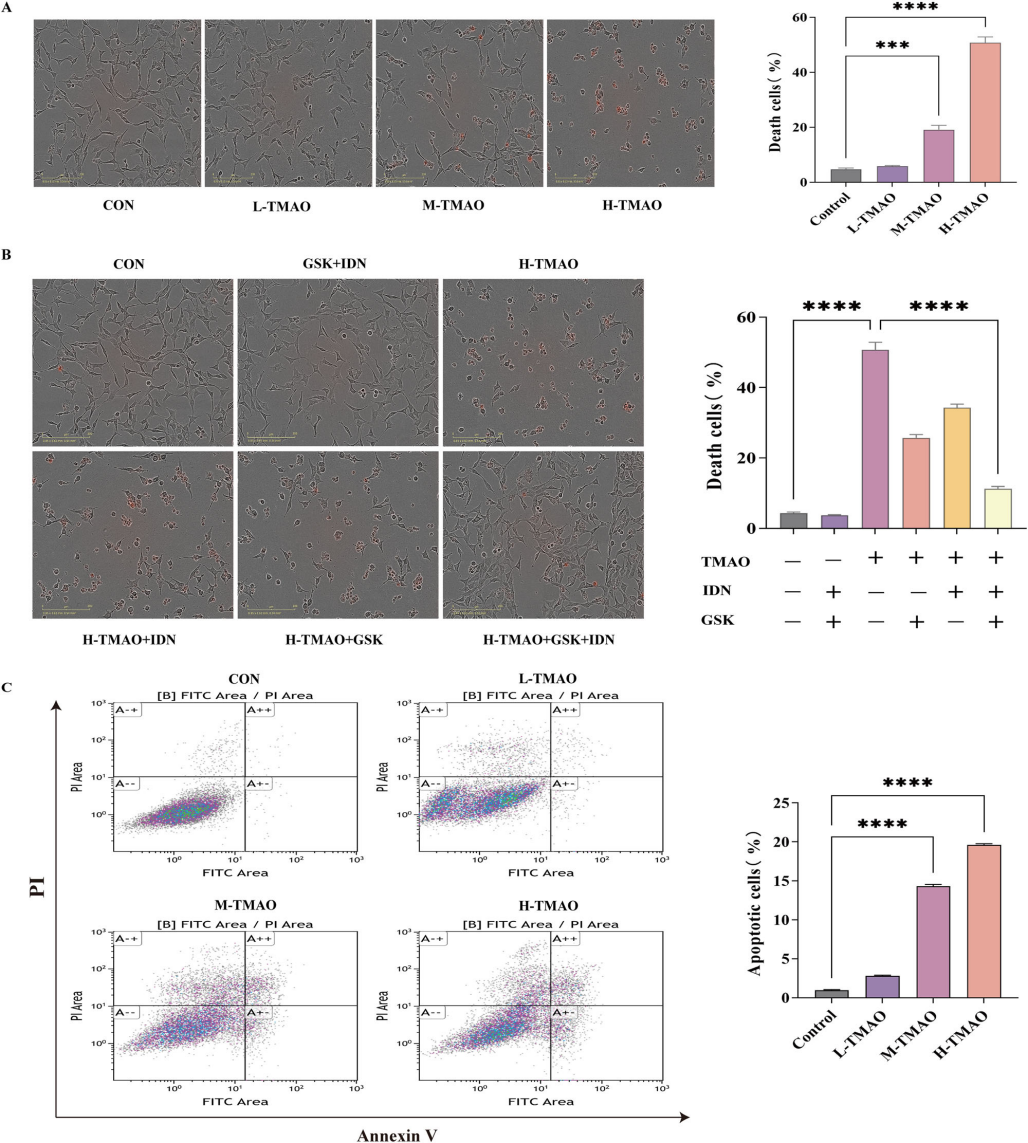
TMAO induces dose-dependent cytotoxicity and apoptosis. **A** Representative images of cell morphology and propidium iodide-positive (PI⁺) dead cells in control, L-TMAO, M-TMAO, and H-TMAO groups. Quantification of dead cell percentage is shown on the right. **B** Representative images and quantification of PI⁺ dead cells in cells treated with H-TMAO alone or in combination with the pan-caspase inhibitor IDN, Ripk3 inhibitor GSK, or both. Control and inhibitor-only groups are also shown. **C** Flow cytometric analysis of apoptosis using Annexin V-FITC/PI staining in control and TMAO-treated groups. Quantification of apoptotic cells is shown on the right. Data analyzed by one-way ANOVA, represented by Mean with SEM, *P < 0.05, **P < 0.01, ***P < 0.001, ****P < 0.0001, *n* = 3 per group.

To investigate the cytotoxic effects of TMAO at varying concentrations, cell viability and apoptosis assays were performed. As shown in Fig. 6A, treatment with increasing concentrations of TMAO resulted in a dose-dependent increase in cell death, as visualized by morphological changes and red-stained dead cells under light microscopy. Quantification revealed a significant elevation in the proportion of dead cells in the M-TMAO and H-TMAO groups compared to the control group, with the H-TMAO group exhibiting the highest level of cytotoxicity.

To further explore the underlying mechanisms, inhibitors targeting specific signaling pathways were used in combination with H-TMAO treatment (Fig. 6B). Co-treatment with GSK-872 (a Ripk3 inhibitor), Emricasan (IDN, a pan-caspase inhibitor), or both markedly reduced cell death induced by H-TMAO, with the combination of GSK-872 and IDN showing the most pronounced protective effect. To investigate the cytotoxic effects of TMAO at varying concentrations, cell viability and apoptosis assays were performed.

Apoptosis was further evaluated by Annexin V/PI staining and flow cytometric analysis (Fig. 6C). Consistent with cell death results, the percentage of apoptotic cells increased in a concentration-dependent manner, with significantly higher apoptosis observed in M-TMAO and H-TMAO-treated groups relative to control cells. These findings indicate that TMAO induces apoptosis in a dose-dependent fashion.

### TMAO induces the assembly and co-localization of the core PANoptosome complex

The PANoptosome complex is a key protein complex formed during the process of PANoptosis, serving as a core platform for the crosstalk between pyroptosis, apoptosis, and necroptosis signaling pathways. The PANoptosome complex is primarily composed of the following core proteins: apoptosis-related protein: Caspase-8; necroptosis-related proteins: RIPK1, RIPK3, MLKL; pyroptosis-related proteins: ASC, NLRP3, Caspase-1; regulatory proteins: ZBP1, STAT3. It is currently believed that the co-localization of Caspase-8, RIPK3, and ASC suggests the formation of the PANoptosome complex.

**Fig. 7:**
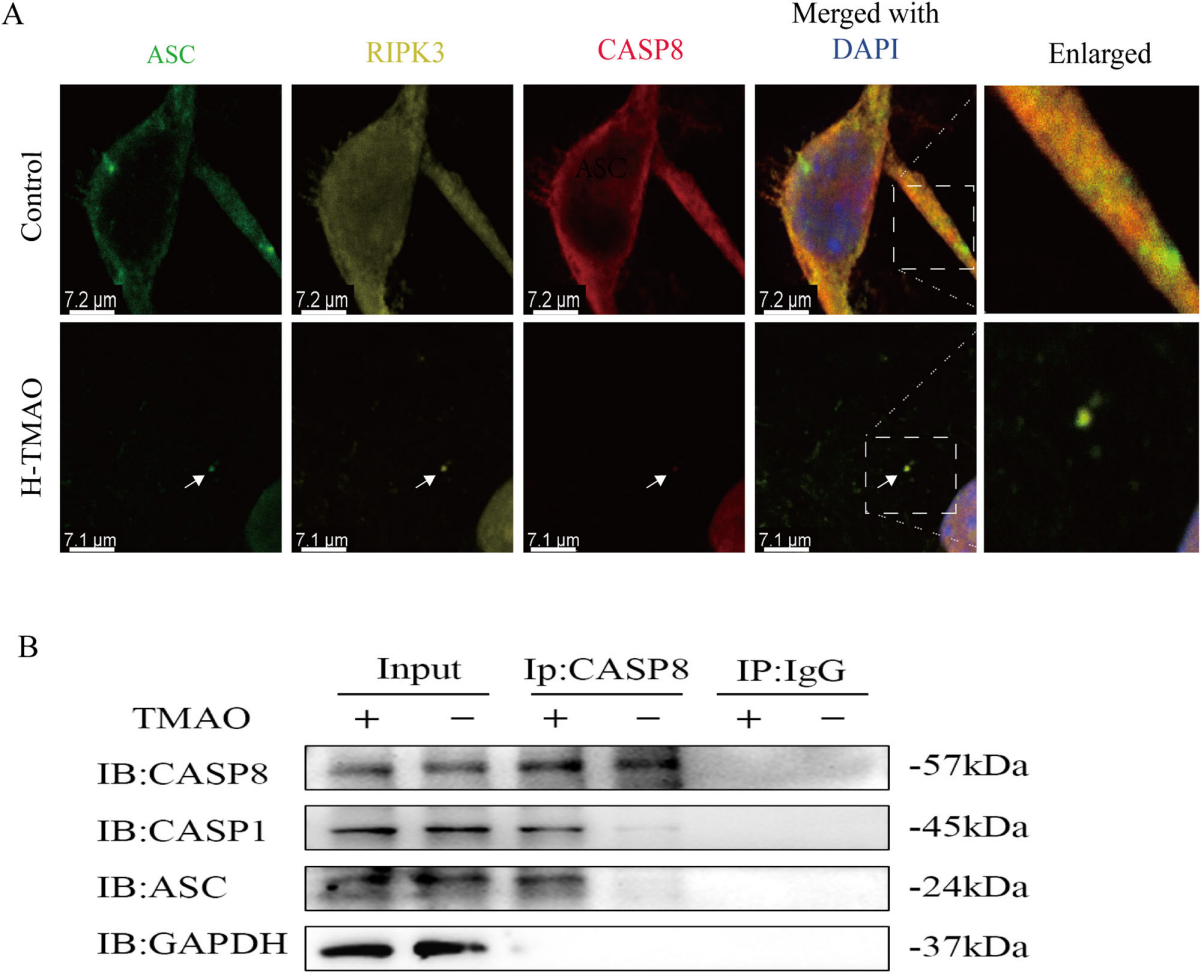
TMAO induces the assembly and co-localization of the core PANoptosome complex. **A** Immunofluorescence analysis of cells treated with or without H-TMAO (Control). Cells were stained for endogenous ASC (green), RIPK3 (yellow), and CASP8 (red). Nuclei were counterstained with DAPI (blue). In control cells, the three proteins showed a diffuse cytoplasmic distribution. Upon H-TMAO treatment, ASC, RIPK3, and CASP8 aggregated to form a single, bright, co-localized speck-like structure (indicated by white arrows). Scale bars: 7.1, 7.2 μm. **B** Co-immunoprecipitation (Co-IP) analysis. Cell lysates from cells treated with (+) or without (-) TMAO were immunoprecipitated (IP) with an anti-CASP8 antibody or control IgG. The immunoprecipitates and input lysates were then subjected to immunoblotting (IB) with the indicated antibodies (CASP8, CASP1, ASC, and GAPDH).

To determine whether trimethylamine N-oxide (TMAO) induces the formation of the PANoptosome, the subcellular localization of its core components—ASC, RIPK3, and Caspase-8 (CASP8)—was first examined by immunofluorescence microscopy. In untreated control cells, these three proteins exhibited a diffuse cytoplasmic distribution (Fig. 7A, upper panel). Following treatment with H-TMAO, however, a marked subcellular reorganization of these proteins was observed. ASC, RIPK3, and CASP8 aggregated to form a single, dense, punctate structure, or speck. Merged image analysis confirmed a high degree of co-localization of the three proteins within this structure (Fig. 7A, lower panel), indicating that TMAO induces their spatial recruitment into a common locale.

To further investigate the physical basis for this co-localization, protein-protein interactions were assessed via co-immunoprecipitation (Co-IP). Immunoprecipitation of endogenous CASP8 from cell lysates revealed no detectable association with either Caspase-1 (CASP1) or ASC in the absence of TMAO stimulation. In contrast, a significant interaction between CASP8 and both CASP1 and ASC was evident in the TMAO-treated samples (Fig. 7B). The specificity of this interaction was established by the failure of a control IgG antibody to immunoprecipitate the target proteins and the absence of the cytosolic protein GAPDH in the CASP8 immunoprecipitates.

Collectively, the imaging and biochemical data support the conclusion that TMAO promotes the physical association of core PANoptosome components, driving their assembly into a multi-protein functional complex.

### TMAO treatment increases the expression of PANoptosis-related proteins in HT-22 neuron cells

**Fig. 8:**
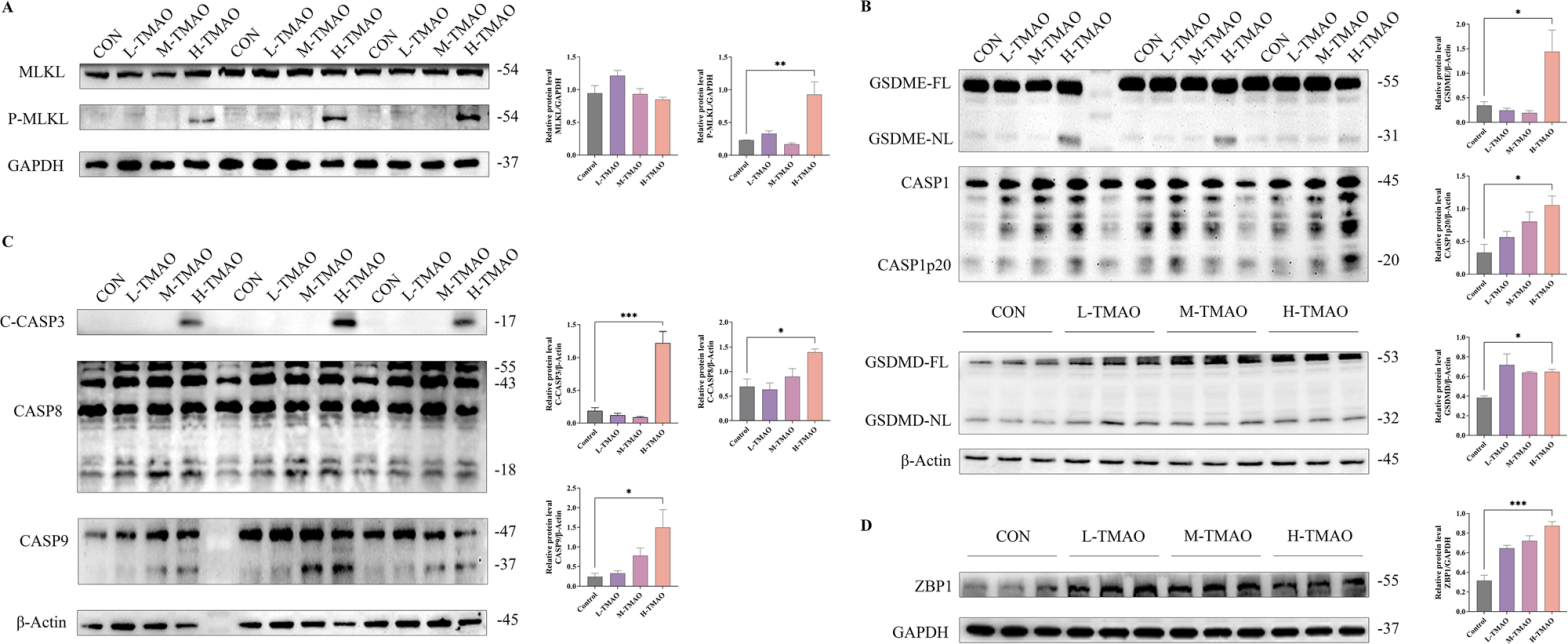
Protein expression of key genes for PANoptosis. **A** Expression of necroptosis-related proteins (MLKL, P-MLKL) in Control, L-TMAO, M-TMAO and H-TMAO groups. **B** Expression of pyroptosis-related proteins (CASP1, GSDMD, GSDME) in Control, L-TMAO, M-TMAO and H-TMAO groups. **C** Expression of apoptosis-related proteins (CASP3, CASP8, CASP9) in Control, L-TMAO, M-TMAO and H-TMAO groups. **D** Protein expression of Zbp1 in Control, L-TMAO, M-TMAO and H-TMAO groups. Standardization strategies use GAPDH/β-actin. Data analyzed by one-way ANOVA, represented by Mean with SEM, *P < 0.05, **P < 0.01, ***P < 0.001, *n* = 3 per group.

To elucidate the mechanisms of TMAO-induced neuronal death, we assessed the expression of PANoptosis-related proteins in HT-22 neuron cells (Fig. 8). High-dose TMAO significantly upregulated ZBP1 expression, suggesting activation of PANoptosis—a coordinated cell death pathway integrating necroptosis, pyroptosis, and apoptosis. Phosphorylated MLKL (p-MLKL), a hallmark of necroptosis, was notably increased in the H-TMAO group, despite unchanged total MLKL levels, indicating specific activation of the necroptotic cascade.

Meanwhile, pyroptotic markers including cleaved GSDMD and GSDME were elevated in TMAO-treated groups, with the strongest induction in the H-TMAO group. CASP1p20 expression was also increased, supporting inflammasome-mediated pyroptosis. Additionally, cleaved caspase-3 levels were markedly higher, along with elevated caspase-8 and caspase-9, indicating concurrent activation of extrinsic and intrinsic apoptotic pathways. These findings collectively support the role of ZBP1-mediated PANoptosis in TMAO-induced hippocampal neuronal damage.

## Discussion

In this study, we provide the first direct evidence that the gut microbiota-derived metabolite, trimethylamine N-oxide (TMAO), impairs hippocampal neuronal function by activating PANoptosis, an integrated and highly inflammatory cell death pathway. While prior research has primarily linked elevated TMAO to the promotion of neuroinflammation, the precise molecular mechanisms by which it inflicts direct neuronal damage have remained elusive. Our findings address this critical gap by identifying PANoptosis as a core downstream mechanism, thus establishing a clear TMAO-PANoptosis-neurodegeneration axis.

This discovery is best understood within the broader context of the microbiome-gut-brain axis, a complex communication network pivotal in health and disease. The gut microbiome influences the central nervous system largely through its metabolic products, which can enter systemic circulation and modulate the host’s immune response, subsequently affecting neuronal activity^48^. In the context of neurodegenerative disorders, gut dysbiosis is increasingly viewed as a key contributor to the progression of neuroinflammation. Metabolites produced by an altered microbiome are thought to be significant drivers of this pathology^45^. Our study contributes a crucial mechanistic insight to this field by demonstrating how TMAO, a metabolite capable of crossing the blood-brain barrier, acts as a direct effector molecule that instigates a destructive cellular program—PANoptosis—in the hippocampus.This positions TMAO not merely as a biomarker of cardiovascular risk but as a direct neurotoxic agent central to the dialogue between the gut microbiome and neuronal viability.

To clarify the effect of TMAO on neural function, we isolated it from choline and other metabolites and treated mice with different doses of TMAO. Our behavioral assessments revealed that chronic TMAO exposure significantly impairs cognitive function. Mice exhibited marked deficits in working and recognition memory, as demonstrated in the Y-maze and novel object recognition tests, and profound spatial memory impairments in the Morris water maze test. Importantly, these cognitive deficits were not attributable to changes in locomotor activity or anxiety, confirming a specific impact on neural circuits.

Histological analyses revealed the neuropathological basis for these behavioral changes. We observed a significant reduction in neuronal density in the CA1 and CA3 subregions of the hippocampus—areas critical for memory encoding and retrieval. This regional specificity aligns perfectly with the observed cognitive impairments. At the ultrastructural level, transmission electron microscopy showed striking mitochondrial abnormalities, including swelling, cristae disintegration, and membrane disruption. This mitochondrial damage appears to be a key upstream event in TMAO-induced neurotoxicity, leading to the activation of downstream cell death pathways.

Our findings are better understood in the context of emerging research on PANoptosis in other neurodegenerative diseases. In Alzheimer’s disease (AD) models, for instance, PANoptosis is often triggered by pathological protein aggregates like amyloid-beta (Aβ) or tau, which typically activate the NLRP3 inflammasome, a key component of the PANoptosome. More recent studies have also implicated other sensors, such as ZBP1, in response to cellular stress in AD. In contrast to these proteinopathy-driven mechanisms, our study reveals a distinct trigger in the AD model. We found that TMAO, a specific microbial metabolite, initiates PANoptosis primarily through zbp1 sensor. This distinction suggests that while PANoptosis may be a convergent cell death mechanism across multiple neurodegenerative disorders, the upstream signaling pathways can be highly disease-specific. Therefore, our work not only identifies a novel regulator of neuronal PANoptosis but also underscores the importance of developing tailored therapeutic strategies that target these unique upstream triggers.

Building on these observations, we investigated the molecular underpinnings of TMAO-induced neuronal death. In vitro analyses revealed a dose-dependent increase in neuronal cell death and apoptosis, as confirmed by microscopic imaging and flow cytometry. Western blot analysis demonstrated activation of multiple cell death pathways, including necroptosis (evidenced by MLKL phosphorylation), pyroptosis (increased cleavage of GSDME, GSDMD, and CASP1), and apoptosis (activation of caspase-3, -8, and -9). Notably, expression of ZBP1, a master regulator of PANoptosis was significantly upregulated by TMAO. This suggests that TMAO may activate a coordinated PANoptosis response in neurons, thereby amplifying its neurotoxic effects. Pharmacological inhibition of RIPK3 and caspases using GSK-872 and Emricasan(IDN) partially rescued neuronal viability, confirming the functional involvement of PANoptosis signaling.

To provide further evidence supporting the induction of PANoptosis in HT-22 cells by trimethylamine N-oxide (TMAO), we employed immunofluorescence staining to label the core proteins ASC, CASP8, and RIPK3. The immunofluorescence results demonstrated that TMAO-induced ASC foci are highly co-localized with RIPK3 and CASP8, suggesting the formation of the PANoptosome complex. To confirm that this spatial reorganization reflected a true molecular complex, we performed co-immunoprecipitation. These experiments revealed a TMAO-dependent physical interaction between CASP8, ASC, and Caspase-1, an association that was absent in control cells. Taken together, these imaging and biochemical data provide robust evidence that TMAO orchestrates the formation of a unified, functional PANoptosome.

Our findings suggest that mitochondrial damage serves as both a catalyst and an amplifier of PANoptosis. At the molecular level, mitochondrial dysfunction was closely associated with the activation of downstream death programs: cytochrome c release may trigger caspase-9/-3-mediated apoptosis^49–52^; mitochondrial distress signals likely sustain NLRP3 inflammasome activity and promote pyroptosis via GSDMD cleavage^53,54^; while RIPK1-RIPK3-MLKL pathway activation supports necroptotic execution^55^. Moreover, the leakage of mitochondrial DNA into the cytosol may further amplify PANoptosis responses by engaging cytosolic DNA sensors such as cGAS-STING and AIM2, establishing a feedforward loop of inflammation and cell death. Together, these observations position PANoptosis not merely as a byproduct but as a pivotal mechanism linking metabolic stress to neuronal degeneration. Rather than discrete activation of isolated pathways, TMAO exposure orchestrates an integrated death program, emphasizing the necessity of targeting upstream metabolic and inflammatory triggers to mitigate neurodegeneration.

Despite the novel insights provided by our study, several limitations should be acknowledged. First, the dosage of TMAO administered in our animal models, while effective for elucidating a clear neuropathological mechanism and consistent with previous studies, may not directly correspond to the levels derived from a typical human diet. The use of a relatively high, acute dose was a methodological necessity to establish a proof-of-concept and induce a robust phenotype within a feasible experimental timeframe. However, this approach does not fully replicate the chronic, low-level exposure characteristic of human dietary patterns. Future research should focus on the long-term effects of lower, more physiologically relevant TMAO concentrations to better model the human condition. Second, this study focused on the downstream neuropathological effects of TMAO without concurrently analyzing the gut microbiome composition of the experimental animals. TMAO is a gut-derived metabolite, and its systemic levels are intrinsically linked to the composition and metabolic activity of the gut flora. Therefore, we cannot draw direct conclusions about which specific alterations in the gut microbiota may have contributed to the observed phenotypes. Future studies incorporating 16S rRNA or metagenomic sequencing would be invaluable to correlate specific microbial signatures with TMAO-induced neurodegeneration. Integrating such multi-omics data would provide a more holistic understanding of the gut-brain axis in this context. Another limitation of this study is that we did not perform a formal statistical correlation analysis between the degree of neuronal loss in specific hippocampal subfields and the corresponding behavioral deficits. While our histological and behavioral data are strongly consistent and qualitatively support this link, future studies with larger animal cohorts will be necessary to quantitatively establish this structure-function relationship and determine its predictive value.

In addition to directly triggering PANoptosis, the detrimental effects of TMAO on neuronal health may be amplified through its influence on other critical cellular pathways, notably the one involving the anti-aging gene Sirtuin 1 (SIRT1)^56,57^. SIRT1 is a crucial deacetylase with well-established neuroprotective functions, playing a key role in preventing neuronal loss, regulating apoptosis, and promoting cognitive health^58,59^. Excitingly, emerging evidence has established an inverse relationship between TMAO and SIRT1^60^. For instance, studies have shown that higher circulating TMAO levels can aggravate cognitive impairment by downregulating hippocampal SIRT1 expression in models of vascular dementia, and that TMAO promotes inflammatory responses through signaling pathways involving SIRT1 suppression^61^. Future studies investigating the direct interplay between TMAO, SIRT1, and the PANoptosome will be a fascinating and important avenue of research^62^.

This study reveals that chronic exposure to TMAO, a gut microbiota-derived metabolite influenced by diet, triggers a PANoptosis signature in hippocampal neurons, involving the concurrent activation of apoptosis, necroptosis, and pyroptosis pathways. Correspondingly, mice exposed to TMAO exhibited marked cognitive deficits, accompanied by hippocampal neuronal loss and elevated levels of PANoptosis markers such as cleaved caspases, MLKL, and GSDMD. These findings highlight a novel mechanism by which dietary and metabolic factors drive neuronal death and cognitive decline, emphasizing the profound impact of gut-derived metabolites on brain health.

Importantly, this research has significant translational relevance and underscores that dietary regulation and gut microbiota management are tangible strategies to prevent or mitigate cognitive impairment. One highly promising approach involves probiotic strategies to remodel the gut microbiome and thereby reduce TMAO production. An alternative strategy, choline restriction, could also limit the substrate for TMAO synthesis, although this approach must be carefully managed given choline’s essential role in cognitive function.

In conclusion, our work identifies TMAO-induced PANoptosis as a key pathological driver and champions targeting the gut-TMAO-brain axis as a potent therapeutic avenue for preserving cognitive function against metabolic and age-related insults.

## Methods

### Animals and drug treatment

A cohort of 24 male C57BL/6 mice (8 weeks old; 20–25 g) was obtained from a certified facility (Nanjing University of Chinese Medicine, Jiangsu). Mice were acclimated to standardized housing (24 °C, 50–70% humidity, 12-h light/dark cycle) with free access to food and water. Blinded methodologies were applied to all procedures. Post-acclimation (7 days), mice were grouped by fat mass and body weight into three equal-sized experimental arms: control (standard water), LTMAO (1.5% TMAO in water), and HTMAO (5% TMAO in water), administered for 8 weeks. Dietary intake remained unmodified. Sample sizes for animal experiments and TMAO exposure levels were determined based on established protocols and effect sizes reported in previous, similar studies in the field to ensure sufficient statistical power^44,63^.

### Cell culture and drug treatments

HT-22 hippocampal neuron cells, purchased from the Cell Resource Center of the Institute of Basic Medical Sciences, Chinese Academy of Medical Sciences. Culturing of HT-22 cells was done in a 5% CO_2_ humidified incubator at 37 °C, using Dulbecco’s modified Eagle’s medium (DMEM, Gibco, USA) with 10% fetal bovine serum (FBS, Gibco, USA). Phosphate buffer saline (PBS, pH = 7.4) was sourced from BDBIO, while 0.25% trypsin-EDTA came from Gibco. The experimental cells were divided into 4 groups, namely the control group (Control), the low-dose TMAO group (L-TMAO, 5 mg/ml), the medium-dose TMAO group (M-TMAO, 10 mg/ml), and the high-dose TMAO group (H-TMAO, 20 mg/ml).

### Behavioral tests

All behavioral tests were conducted by an experimenter who was blinded to the experimental group assignments to prevent observer bias. To prevent inter-test interference and the confounding effects of stress, all behavioral experiments were conducted in a specific sequence, proceeding from the least to the most stressful paradigm. The testing battery was ordered as follows: the open field test, the Y-maze test for spontaneous alternation, the novel object recognition test, and finally, the Morris water maze. A minimum of 24 h separated the lower-stress tests (open field, novel object recognition and Y-maze). A longer rest period of 48 h was provided after the completion of the novel object recognition test and before commencing the Morris water maze protocol to ensure a full recovery to a baseline state. All tests were performed during the light phase of the light/dark cycle, and all apparatuses were thoroughly cleaned with 70% ethanol between subjects to eliminate olfactory cues.

#### Open field test

Mice were carefully positioned in a corner of the open-field apparatus (40 cm × 40 cm × 35 cm) to assess locomotion and anxiety without inducing impairments. The mice were allowed 5 min of unrestricted movement, with their activity automatically monitored via a video camera connected to the ANY-Maze tracking system. The software ANY-Maze was used to analyze the time spent in the central area, total distance traveled, and average speed.

#### Y-maze test

A standardized Y-maze apparatus (inter-arm angle 120° ± 2°) with frosted acrylic material was used to prevent visual cue interference. Each arm channel dimensions: length 40 cm, width 6 cm, side wall height 10 cm. During the training session, the animal spent 5 min exploring the maze with just two arms accessible (the starting arm and another arm), following a 1-h break between trials. For a 10-min test trial, the animal was situated in the Y-maze with access to all three arms, including the novel arm. In the course of testing, the quantity of entries into three arms served as a metric for motor performance, arm entries, and triads. After each animal is tested, wipe the maze with alcohol to avoid residual odors interfering with subsequent experiments. Using the ANY-Maze animal video tracking system, the time spent in the new arm was tracked for additional analysis. The alternation rate is calculated using the formula: Alternation % = (N−2)/(T−2) × 100%, where N represents the actual number of alternations and T denotes the total number of arm entries. A valid alternation is defined as three consecutive entries into different arms^64^.

#### Novel object recognition test (NORT)

NORT is a behavioral assessment used to evaluate an animal’s memory and cognitive function by measuring its ability to recognize a new object in its environment. The Novel Object Recognition Test (NORT) was used to assess short-term cognitive functions in mice, focusing on learning and memory, by utilizing their natural preference for novel stimuli. Conducted over a single day, the protocol comprised two phases. Prior to testing, mice underwent habituation in the experimental environment to ensure acclimatization. In the first training session, mice were allowed to explore two identical objects for 5 min. Following a 1-h interval in their home cages, the test phase commenced, wherein one object was substituted with a novel counterpart. Mice interacted with both objects for 3 min, and exploration times for familiar and novel objects were measured using ANY-Maze video tracking software.

#### Morris water maze test (MWM)

Standardized water maze apparatus (diameter 1.5 m, wall height 0.6 m) equipped with a PID temperature control module (accuracy ±0.5 °C) to maintain a water temperature range of 24–26 °C. The spatial navigation area is divided into four 90° sectors by an orthogonal coordinate system. The hidden platform (radius 5 cm) is located 30 cm from the pool wall in a specific quadrant, with the platform surface 10 mm below the water surface. Before the test, a visible platform test was conducted to rule out any confounding effects from non-memorial deficits, such as differences in visual acuity, motivation, or swimming ability. For this trial, a brightly colored flag was attached to the platform, making it clearly visible above the water’s surface. Each mouse was subjected to a single 60-s trial, ensuring no significant sensorimotor differences existed between the experimental groups. Following the exclusion of mice unable to acclimate to the aquatic environment, the remaining mice underwent a 5-day water maze training protocol to assess spatial learning. Daily sessions (10:00 AM–3:00 PM) comprised two trials spaced 30 min apart. Mice were released from alternating quadrants facing the pool wall in each trial and given 60 s to find a submerged platform. Animals unable to locate the platform within the allotted time were manually directed to it. On day 6, a 60-s probe trial was conducted with the platform removed, during which platform crossings were quantified. Parameters such as path trajectory, escape latency, target quadrant dwell time and crossing frequency were systematically monitored and analyzed using the ANY-Maze video tracking software.

### Animal anesthesia and euthanasia

All animal experiments were conducted in strict accordance with the guidelines of the Institutional Animal Care and Use Committee (IACUC) of Nanjing University of Chinese Medicine and were approved by the committee.

Prior to all related experimental procedures, C57BL/6 mice were anesthetized via isoflurane inhalation. Anesthesia was induced in an induction chamber with 3–4% isoflurane mixed with 100% oxygen at a flow rate of 1 L/min. Once the animal lost consciousness and exhibited stable respiration, it was transferred to a surgical station and anesthesia was maintained with 1.5–2.5% isoflurane delivered via a nose cone. The depth of anesthesia was monitored throughout the procedure by assessing the respiratory rate and the absence of the pedal withdrawal reflex. To prevent hypothermia, the animal’s body temperature was maintained at 37 ± 0.5 °C using a homeothermic heating pad.

At the conclusion of the experiments, animals were euthanized using methods approved by the American Veterinary Medical Association (AVMA). Animals were first deeply anesthetized by inhaling a high concentration of isoflurane (5%) in a chamber until respiration ceased. Isoflurane exposure was continued for at least 2 min following respiratory arrest to ensure deep unconsciousness. Cervical dislocation was then performed as a secondary physical method to ensure immediate death. Death was confirmed by observing the absence of heartbeat, fixed and dilated pupils, and the complete cessation of respiration for over 5 min.

### Hippocampal tissue immunofluorescent staining

The mice were transcardially perfused with cold saline and then 4% paraformaldehyde after the behavior tests. Brain tissues were removed and then fixed overnight at 4 °C in 4% paraformaldehyde, and then immersed in 20% and 30% sucrose solutions for 24 h each. The brains were then embedded in optimal cutting temperature compound (OTC Compound, Sakura, USA). The sample dish containing OCT compound and tissue is placed into liquid nitrogen to rapidly freeze. After removing the tissue ice blocks, place them in a −80 °C refrigerator for storage and reserve. Tissue blocks were cut into 20-μm sections using a Cryostat microtome. Frozen sections underwent 20-min permeabilization with 0.4% Triton X-100. Following three PBS buffer washes (0.01 M, pH 7.4), gradient microwave heat repair was conducted using a citrate buffer. During the blocking stage, sections were incubated for 1 h at RT in a solution of 3% BSA and 0.3% Triton X-100. Subsequently, the sections were treated with a primary antibody solution containing Neun (1:500) and incubated overnight at 4 °C. This was followed by three washes with PBS buffer (0.01 M, pH 7.4). The sections were then incubated in the dark with Alexa Fluor 647 secondary antibodies (anti-mouse, 1:500) for 1 h, triple-washed with PBS buffer, and mounted with antifade mounting agent containing DAPI. Images were collected using a fluorescence scanning system, and ImageJ was used for image analysis. For all histological analyses, slides were coded prior to imaging so that the investigator performing the quantification was blinded to the treatment conditions. The codes were not revealed until the analysis was complete.

### Transmission electron microscopy (TEM) observation

Following dissection, hippocampal specimens were triple-washed in PBS (0.1 M, 15 min per cycle) and fixed for 24 h in a dual-aldehyde solution (2% paraformaldehyde, 2.5% glutaraldehyde). Tissues were subsequently post-fixed with 1% osmium tetroxide in 0.1 M phosphate buffer (PB, pH 7.4, 2 h, RT). After PB rinses, dehydration proceeded through an ascending ethanol gradient (50–100%, 15 min per step) and acetone substitution (two 15-min intervals). Infiltration utilized EPON812-acetone ratios (1:1, 4 h; 2:1, overnight at 4 °C), transitioning to undiluted EPON812 (8 h). Polymerization involved sequential curing (37 °C overnight, 60 °C for 48 h). Sections (70 nm) were contrast-stained (uranyl acetate/lead citrate) and visualized via TEM (JEM-2100, 100 kV). Morphometric analysis of subcellular structures was conducted using ImageJ.

### Live cell imaging

The HT-22 mouse hippocampal neuron cells were cultured to an appropriate state and seeded into 24-well plates, with 1 × 10^5^ cells per well. After cell seeding, the culture plate was gently rotated several times to evenly distribute the HT-22 cells. The cells were incubated overnight in a 37 °C, 5% CO_2_ humidified incubator. Following overnight culture, the medium was discarded, and cells were rinsed with 1 ml of pre-warmed 37 °C PBS per well. After removing the PBS wash solution, wells were grouped and 0.5 ml of medium with varying TMAO concentrations was added to induce apoptosis in the cells. Propidium iodide (PI) dye at a concentration of 20 nM was introduced to each well to assess membrane permeability, and the plate was subsequently placed in the IncuCyte incubation imaging system. The culture plate was imaged every hour using a 20× objective per well.

### Flow cytometry

The HT-22 mouse hippocampal neuron cells were cultured to an appropriate state and inoculated into T25 cell culture flasks, with 1 × 10^6^ cells inoculated per flask. They were incubated overnight in a 37 °C, 5% CO_2_ humidified incubator. After overnight culture, the culture medium was removed, and 3 ml of 37 °C warm PBS was added to each flask to wash the cells. The PBS wash solution was removed, the culture flasks were grouped, and 5 ml of culture medium containing different concentrations of TMAO was added to stimulate the cells to initiate apoptosis. After the TMAO stimulation period is over, the medium is removed and 3 ml of 37 °C warm PBS is added to each bottle to wash the cells. Treat the cells with trypsin for 1 min, stop the digestion process, centrifuge the cell suspension at 1000 rpm for 5 min, remove the supernatant, and wash the cells twice with PBS that has been pre-cooled to 4 °C. Perform centrifugation at 1000 rpm for 5 min, retrieve the cells, introduce 100 µl of binding buffer, and gently pipette to resuspend them. Add 5 µl of Annexin V-488 and mix gently by pipetting. Subsequently, add 5 µl of PI, mix gently once more, and incubate in the dark at RT for 5–15 min. After incubation, add 400 µl of binding buffer, gently pipette to mix, keep the mixture in the dark, and detect with a flow cytometer.

### HT-22 immunofluorescent staining

The cellular slides were subjected to a series of preparatory steps beginning with washing in PBS. Subsequently, samples were fixed with 4% paraformaldehyde at RT for 20 min. Cells were permeabilized using 0.3% Triton X-100 in PBS for 15 min. Subsequently, blocking was conducted with 3% BSA at 25 °C for 30 min. The labeling protocol involved three steps: first, incubation with the primary antibody in a humidified chamber at 4 °C for 16 h with humidity above 90%; second, incubation with a 1:500 diluted HRP-conjugated secondary antibody at 25 °C for 50 min; and third, development with a 1:50 diluted iF488-TSA in the dark for 10 min. Epitope regeneration was facilitated by antibody elution using G1266 elution solution, initially at RT for 5 min followed by 30 min at 37 °C. Cyclic labeling was executed using iF555-TSA for the Cy3 channel and iF647-TSA for the Cy5 channel, concluding with a final nuclear staining using DAPI for 10 min and mounting with an anti-quenching agent. Multispectral imaging was conducted utilizing a Nikon A1R confocal microscope.

### Co-immunoprecipitation (Co-IP)

HT22 cells were treated with 20 mg/mL trimethylamine N-oxide (TMAO) for 16 h. After stimulation, cells were washed twice with ice-cold PBS and lysed in RIPA buffer (NCM Biotech, China) supplemented with 1 mM PMSF and a protease inhibitor cocktail on ice for 30 min. Cell lysates were centrifuged at 12,000 × *g* for 15 min at 4 °C, and the supernatants were collected. Protein concentrations were quantified using a BCA Protein Assay Kit (Thermo Fisher Scientific). For immunoprecipitation, 500 µg of total protein was incubated overnight at 4 °C with 2 µg of rabbit anti-caspase-8 antibody (Proteintech) or normal rabbit IgG (control). The immune complexes were captured by incubation with 30 µL of protein A/G magnetic beads (Thermo Fisher Scientific) for 2 h at 4 °C with gentle rotation. Beads were washed five times with ice-cold lysis buffer, and bound proteins were eluted by boiling in SDS sample buffer for 10 min. Immunoprecipitates and input lysates were analyzed by Western blotting using antibodies against GAPDH, Caspase-8, Caspase-1, and ASC.

### Western blot analysis

The levels of p-MLKL, MLKL, Caspase-1, GSDMD, GSDME, Cleaved Caspase-3, Caspase-8, Caspase-9, β-actin, and GAPDH were assessed in both the hippocampus of mice and HT-22 mouse hippocampal neuron cells. All antibodies were procured from Cell Signaling Technology (CST, USA), with the exception of β-actin, Caspase-8, Caspase-9 and GAPDH, which were sourced from Proteintech Group (Proteintech, USA). Antibodies were prepared in 1% BSA at a 1:1000 dilution, except for β-actin and GAPDH, which were diluted at 1:5000. The whole hippocampus and HT-22 cells were lysed in RIPA buffer with protease and phosphatase inhibitors. Protein concentrations were determined using the colorimetric method with the NANODROP 2000 (Thermo Scientific, USA). Proteins were resolved using 8–12% SDS-PAGE and subsequently transferred to poly vinylidene difluoride (PVDF) membranes. Following a 1.5 h blocking step with 3% bovine serum albumin (BSA) at ambient temperature, membranes were probed with primary antibodies at 4 °C overnight. Subsequently, horseradish peroxidase (HRP)-linked secondary antibodies were applied for 2 h. Immunoreactive bands were detected using the SuperSignal West Pico Chemiluminescent Substrate (Thermo Scientific, USA) within an enhanced chemiluminescence (ECL) system. ImageJ software was used for band quantification. All assays were replicated three times, with normalized data expressed as relative optical density (ROD) values against GAPDH or β-actin controls.

### Statistical analysis

Experimental data are presented as mean ± S.E.M., unless otherwise specified. All graphs were created and statistical analyses were performed using GraphPad Prism 10 (version 10.4.1) and SPSS (version 25.0). Statistical methods were selected based on the experimental design. A single-factor analysis of variance (ANOVA) was used for comparisons among multiple independent groups, while a two-factor ANOVA was employed for multivariable analyses. Prior to performing ANOVA, the assumption of homogeneity of variance was verified using the Brown-Forsythe and Bartlett’s tests, and other prerequisite assumptions for ANOVA were confirmed. When a statistically significant result was found by ANOVA, Tukey’s multiple comparisons test was then used to analyze the differences between groups. For all statistical tests, the significance level was set at *P* < 0.05.

## Supplementary information

Supplementary Information

## Data Availability

The data that support the findings of this study are available from the corresponding author upon reasonable request.
