# Supplementary material for: Dietary choline-derived Trimethylamine N-oxide impairs hippocampal neuronal function via PANoptosis activation
Source: NPJ Sci Food. 2025 Nov 17;9:235. doi: 10.1038/s41538-025-00599-1 (PMC12624029; doi:10.1038/s41538-025-00599-1)
Supplement: Supplementary file 1 — Supplementary Information [file 41538_2025_599_MOESM1_ESM.docx]

*The original date of western bolt*

*
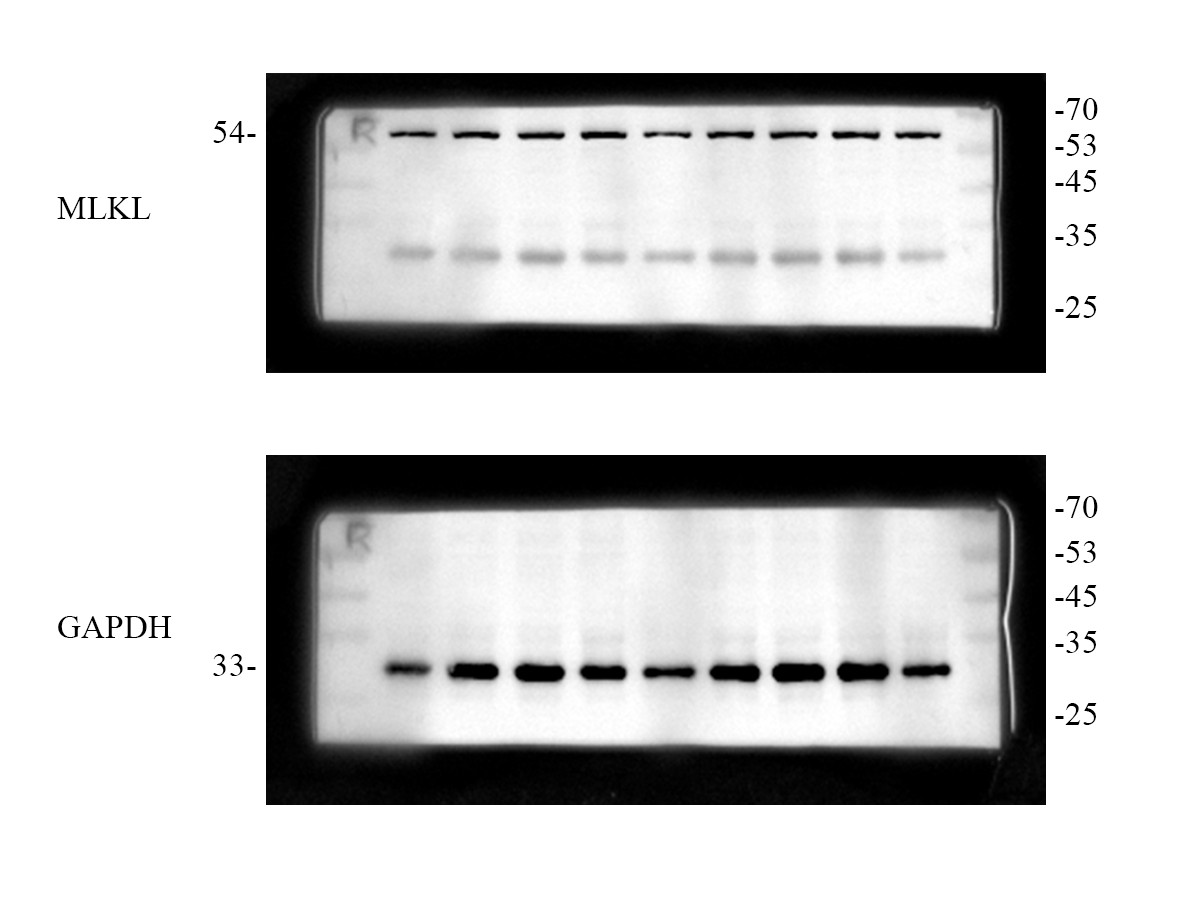
*

**Figure S1**: Uncropped blot images corresponding to Figure 5A MLKL.Lane assignments: Samples from Control (Con), Low-dose TMAO (L-TMAO), and High-dose TMAO (H-TMAO) groups were loaded in an alternating manner for three biological replicates.


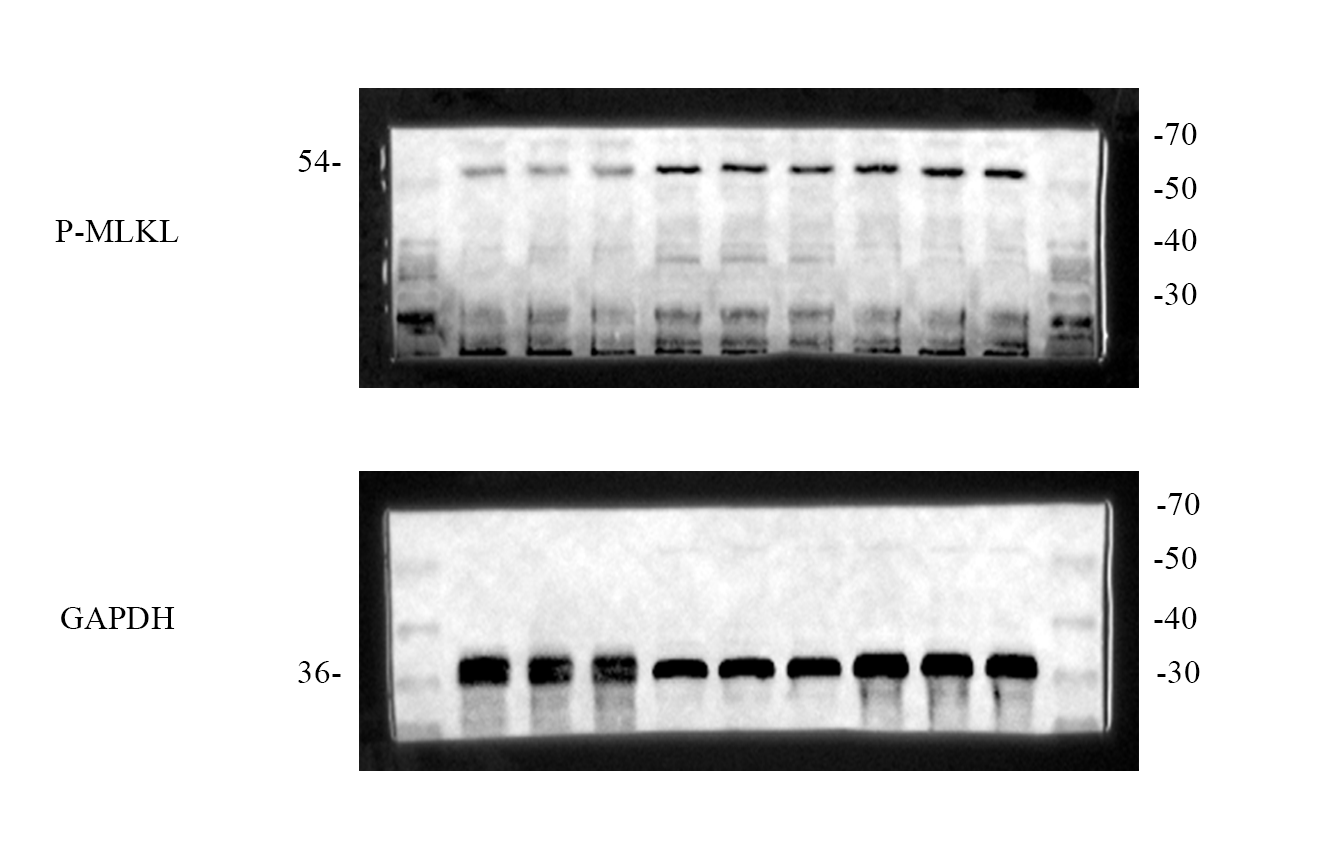


**Figure S2**: Uncropped blot images corresponding to Figure 5A P-MLKL.Lane assignments: Three biological replicates for each group were loaded consecutively. Lanes 1-3: Control (Con); Lanes 4-6: Low-dose TMAO (L-TMAO); Lanes 7-9: High-dose TMAO (H-TMAO).


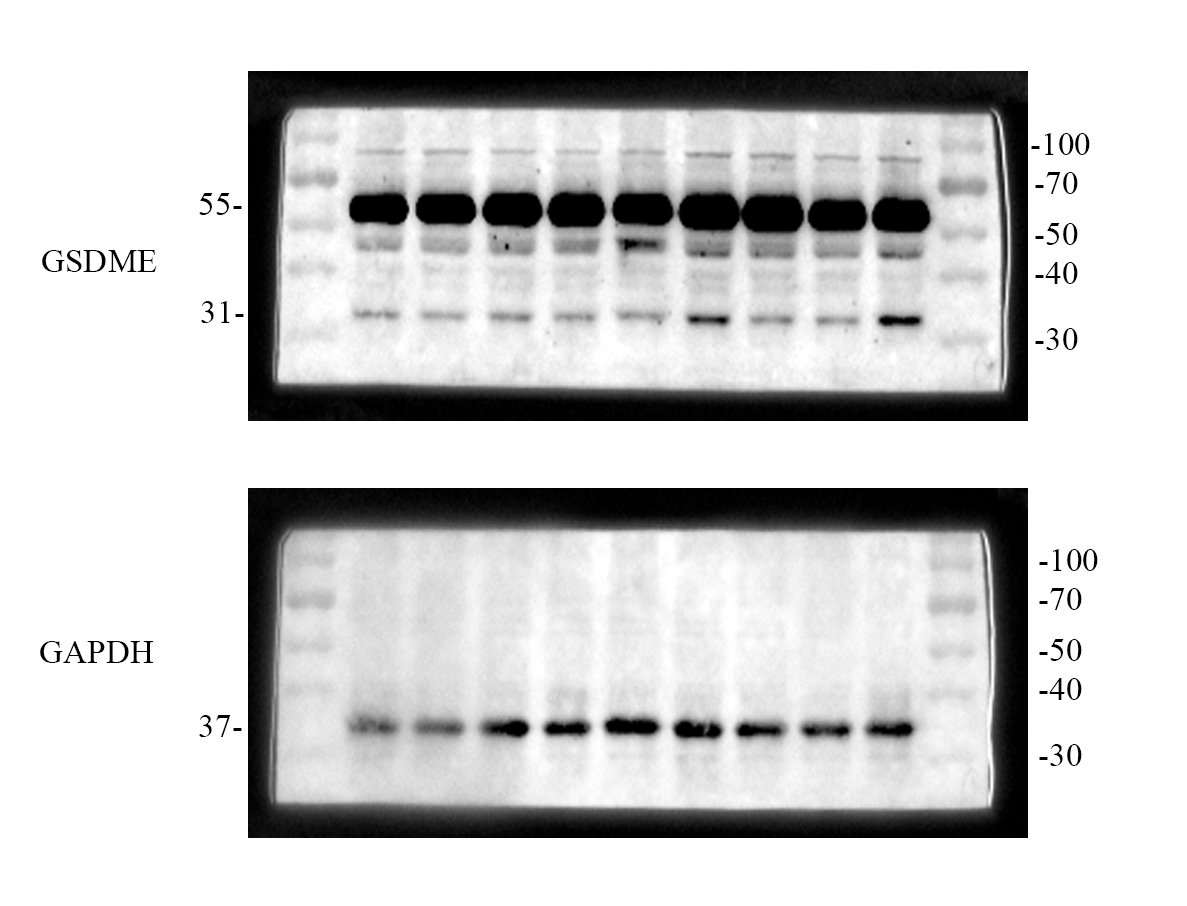


**Figure S3**: Uncropped blot images corresponding to Figure 5B GSDME.Lane assignments: Samples from Control (Con), Low-dose TMAO (L-TMAO), and High-dose TMAO (H-TMAO) groups were loaded in an alternating manner for three biological replicates.


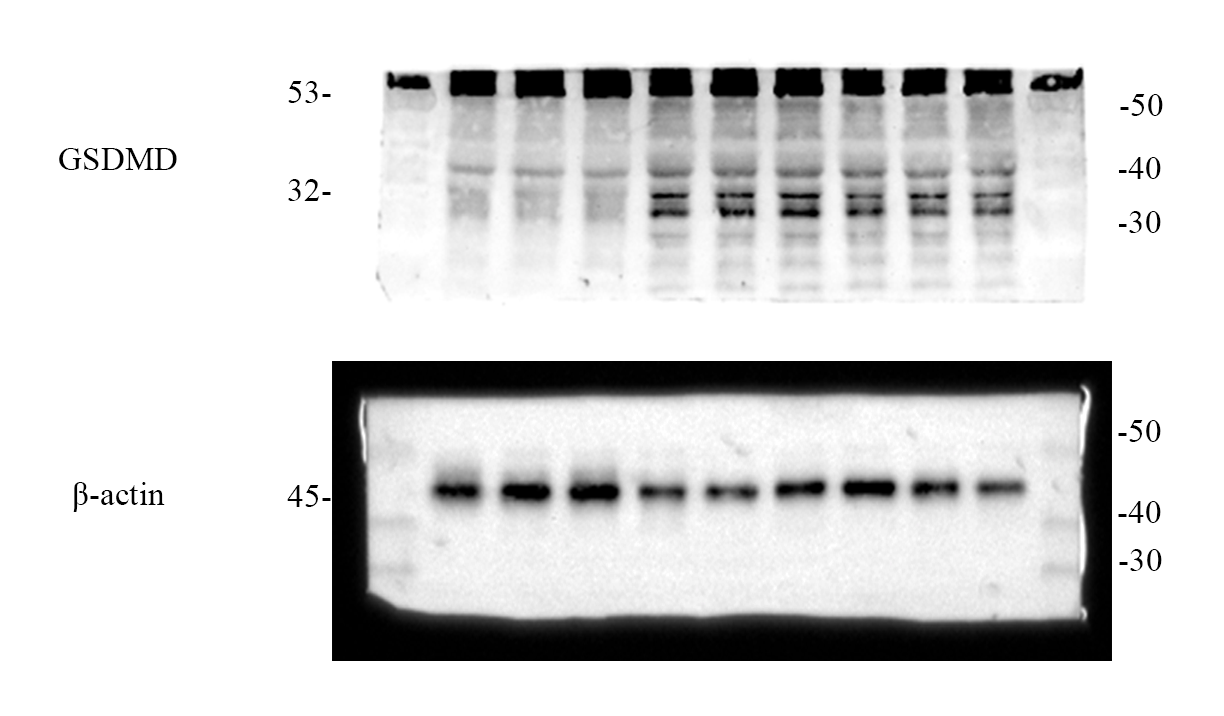


**Figure S4**: Uncropped blot images corresponding to Figure 5B GSDMD.Lane assignments: Three biological replicates for each group were loaded consecutively. Lanes 1-3: Control (Con); Lanes 4-6: Low-dose TMAO (L-TMAO); Lanes 7-9: High-dose TMAO (H-TMAO).


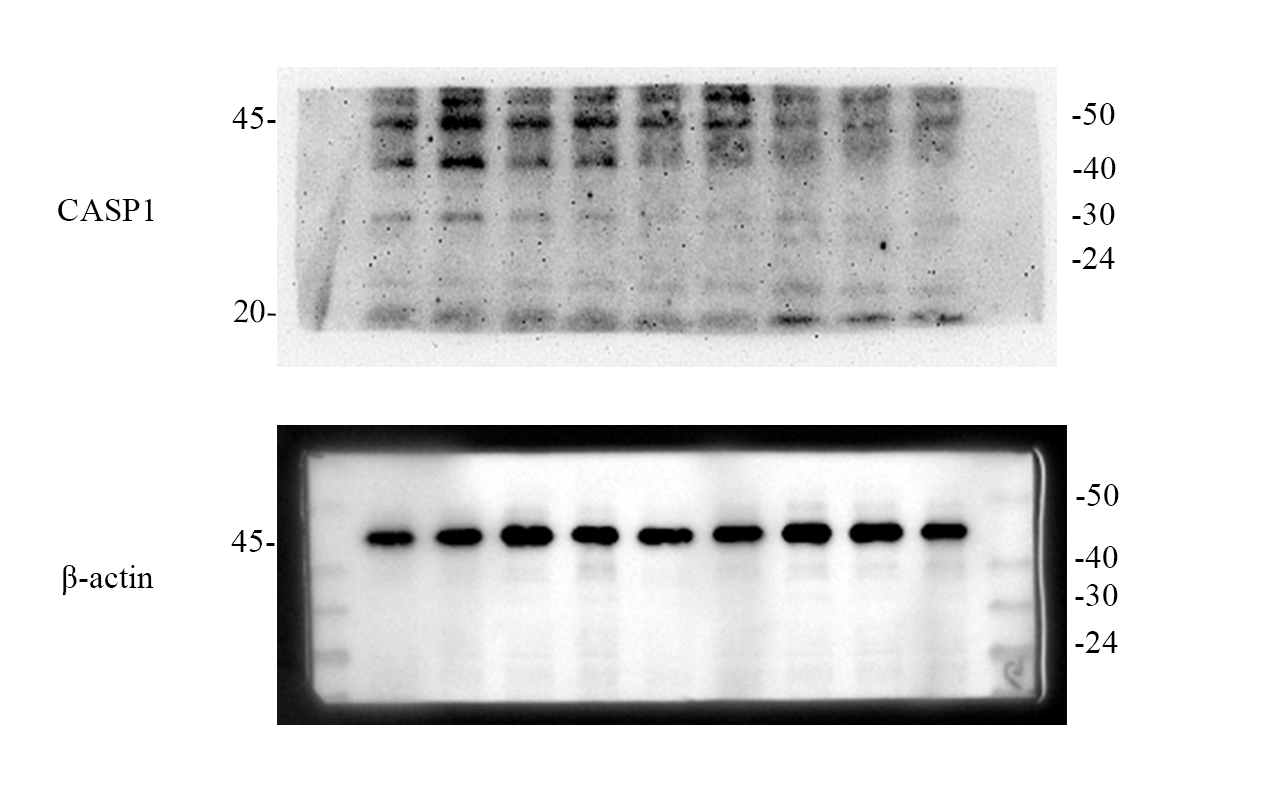


**Figure S5**: Uncropped blot images corresponding to Figure 5B CASP1.Lane assignments: Three biological replicates for each group were loaded consecutively. Lanes 1-3: Control (Con); Lanes 4-6: Low-dose TMAO (L-TMAO); Lanes 7-9: High-dose TMAO (H-TMAO).


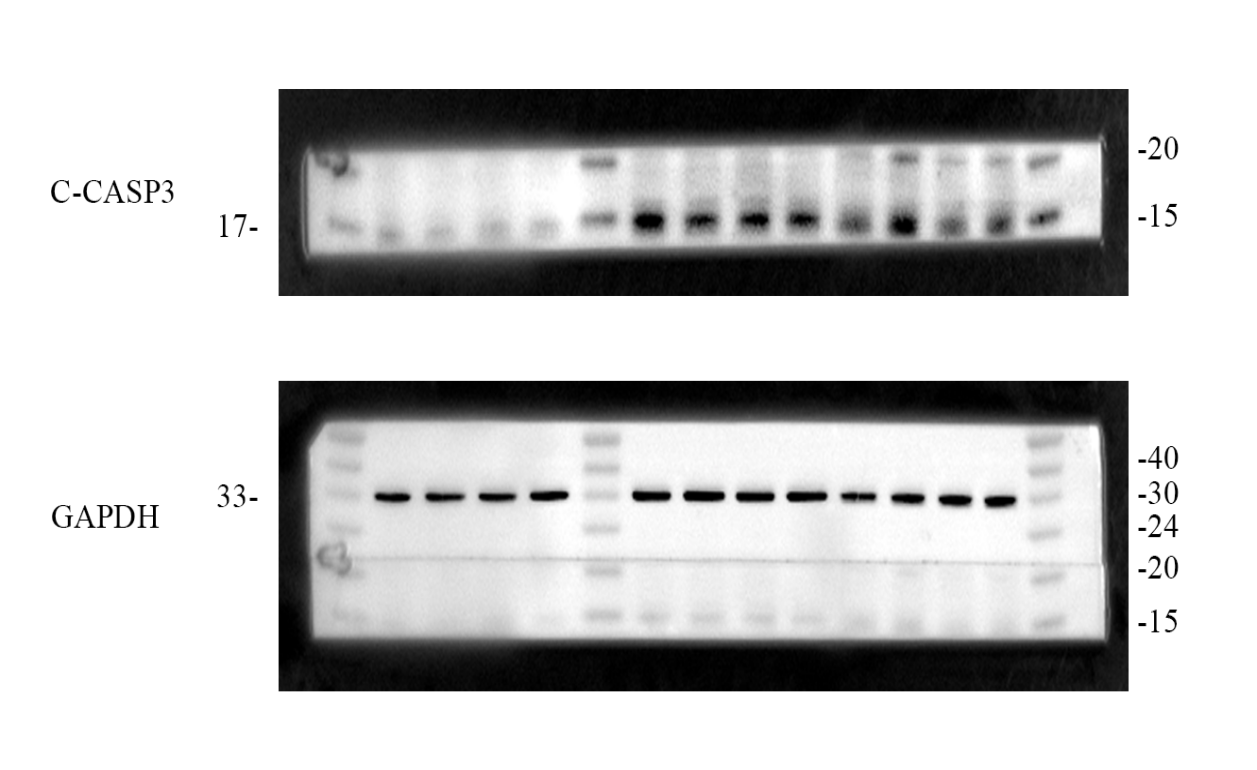


**Figure S6**: Uncropped blot images corresponding to Figure 5C C-CASP3.The same membrane was cut into strips according to the molecular weight markers to allow for simultaneous incubation with antibodies against C-CASP3and GAPDH.Lane assignments: Three biological replicates for each group were loaded consecutively. Lanes 1-4: Control (Con); Lanes 5-8: Low-dose TMAO (L-TMAO); Lanes 9-12: High-dose TMAO (H-TMAO).


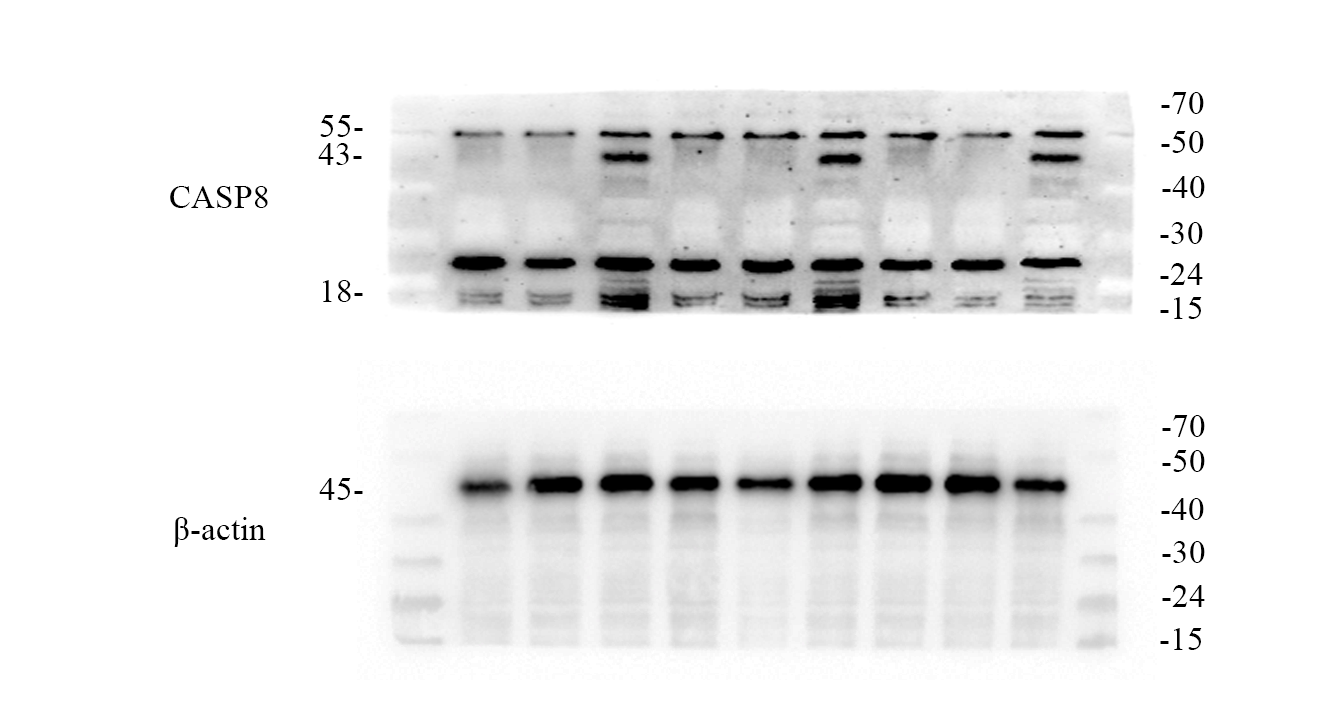


**Figure S7**: Uncropped blot images corresponding to Figure 5C CASP8.Lane assignments: Samples from Control (Con), Low-dose TMAO (L-TMAO), and High-dose TMAO (H-TMAO) groups were loaded in an alternating manner for three biological replicates.


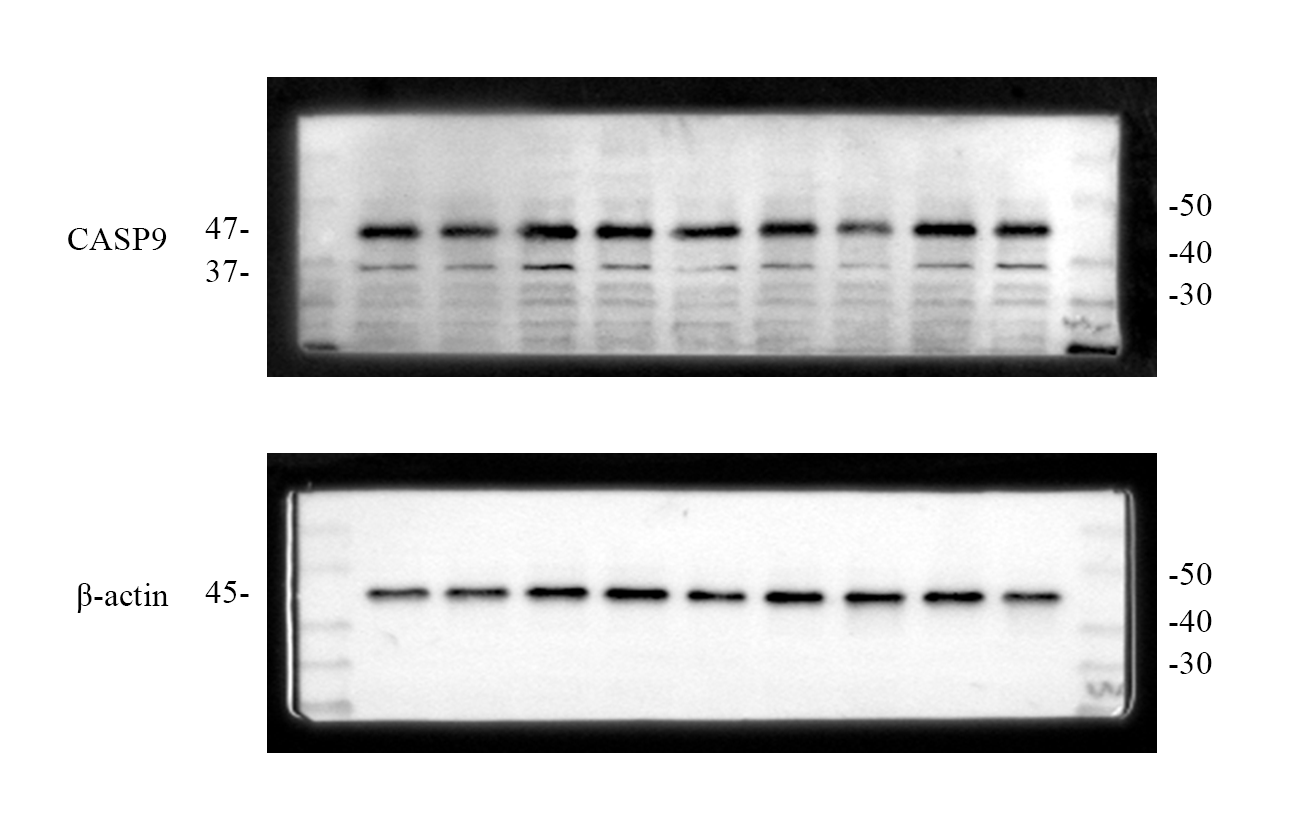


**Figure S8**: Uncropped blot images corresponding to Figure 5C CASP9.Lane assignments: Samples from Control (Con), Low-dose TMAO (L-TMAO), and High-dose TMAO (H-TMAO) groups were loaded in an alternating manner for three biological replicates.


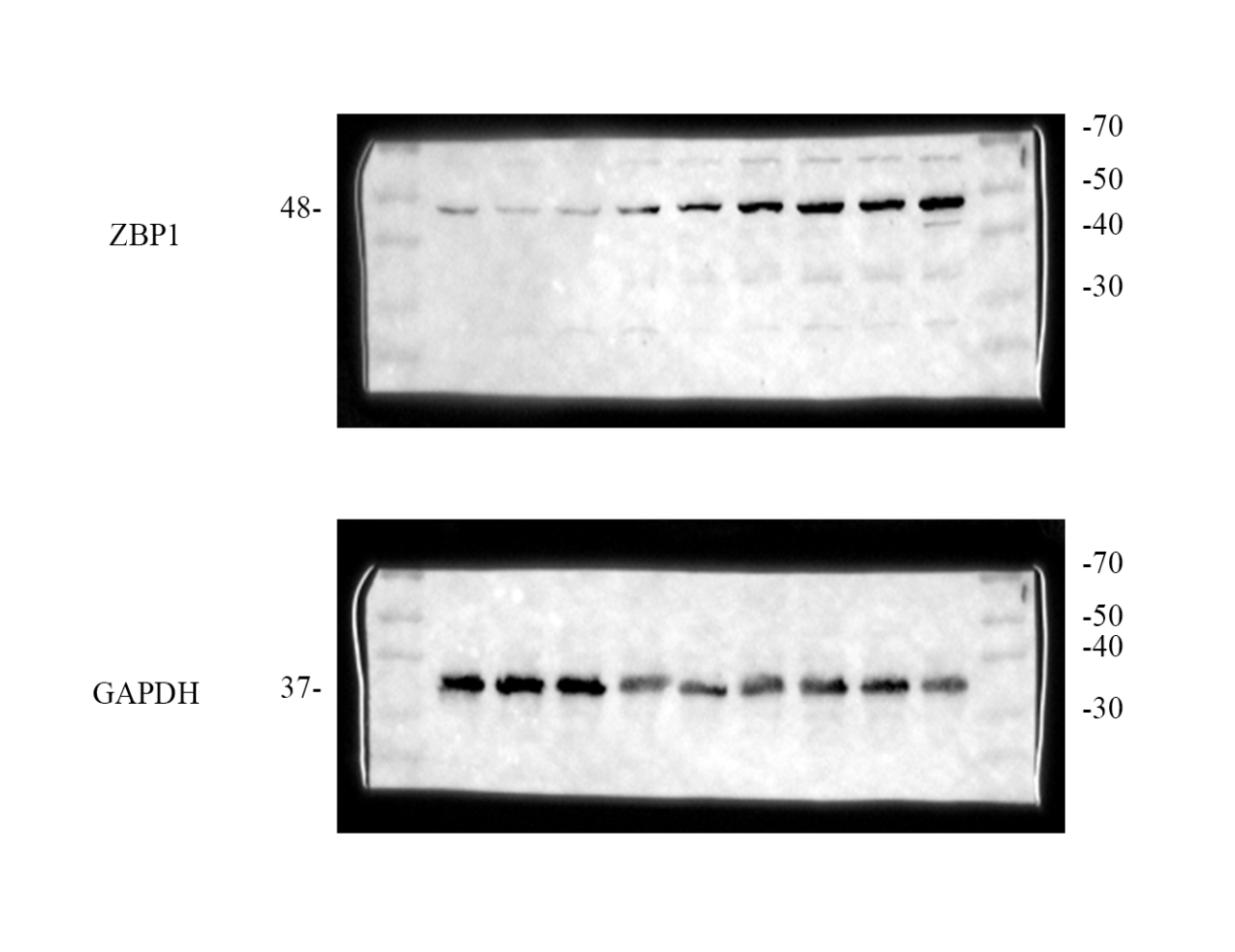


**Figure S9**: Uncropped blot images corresponding to Figure 5D ZBP1.Lane assignments: Three biological replicates for each group were loaded consecutively. Lanes 1-3: Control (Con); Lanes 4-6: Low-dose TMAO (L-TMAO); Lanes 7-9: High-dose TMAO (H-TMAO).


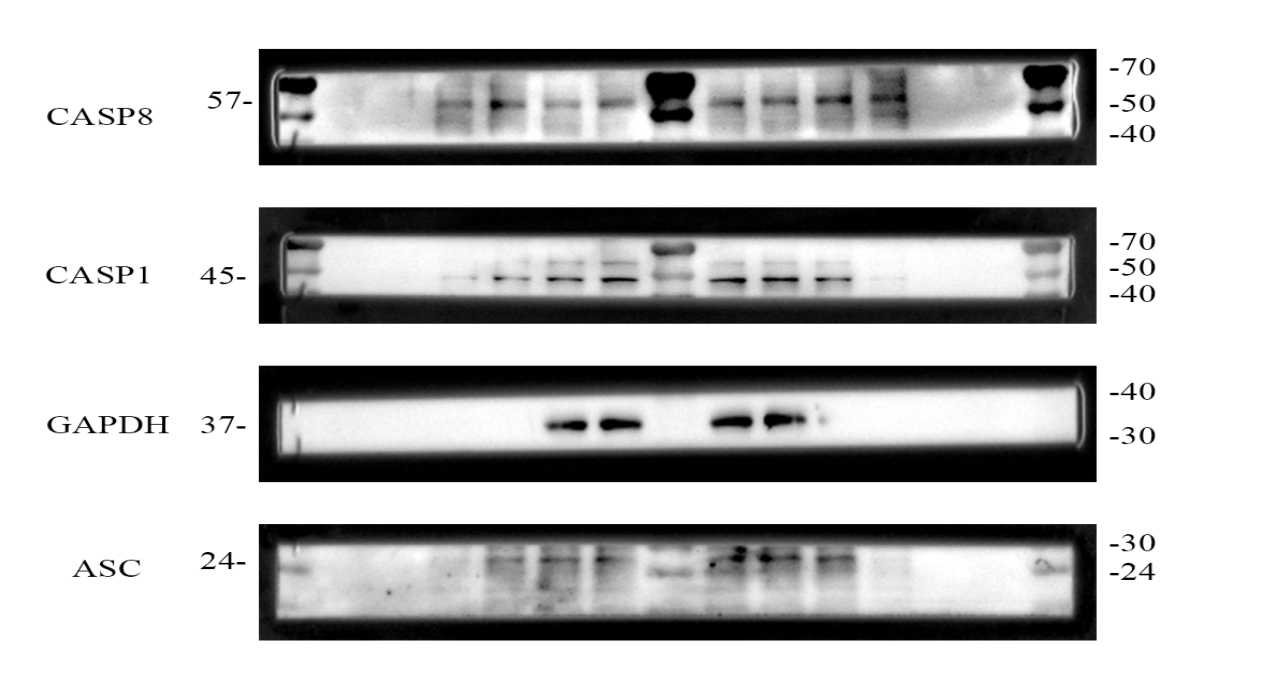


**Figure S10**: Uncropped blot images corresponding to Figure 7B .The lane assignments were arranged symmetrically from the center outwards: lanes 1-2, Input; lanes 3-4, the specific immunoprecipitation antibodies as indicated; lanes 5-6，IgG control.


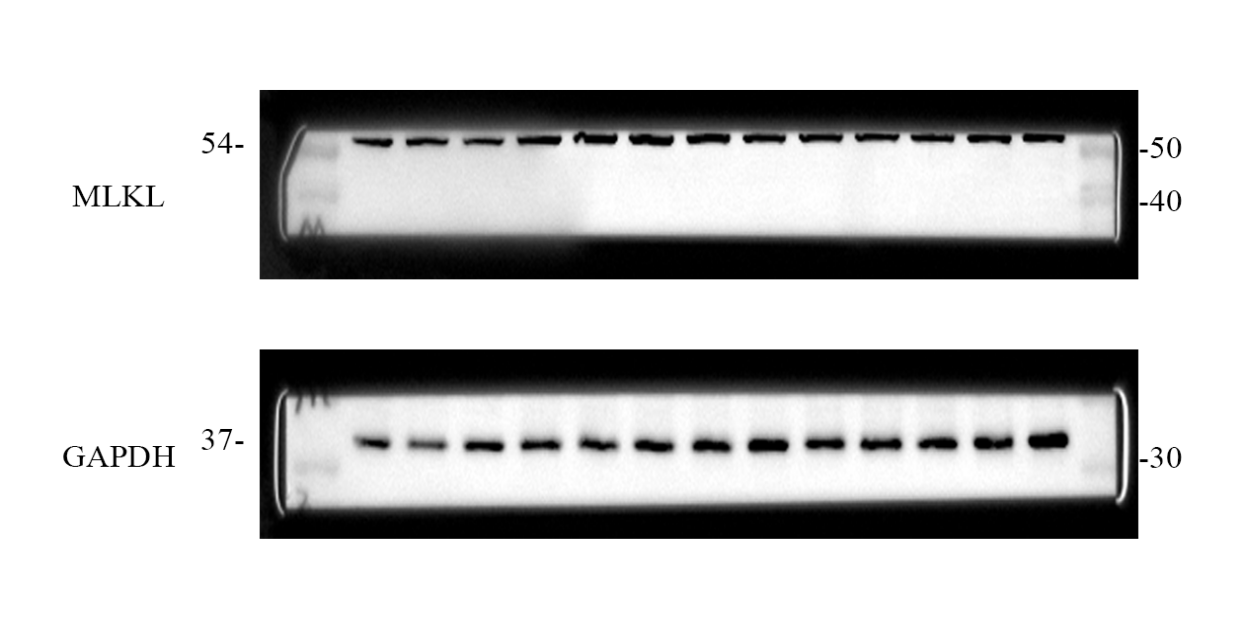


**Figure S11**: Uncropped blot images corresponding to Figure 8A MLKL.The same membrane was cut into strips according to the molecular weight markers to allow for simultaneous incubation with antibodies against MLKL and GAPDH.Lane assignments: Samples from Control (Con), Low-dose TMAO (L-TMAO), Medium-dose TMAO (M-TMAO), and High-dose TMAO (H-TMAO) groups were loaded in an alternating manner for three biological replicates.


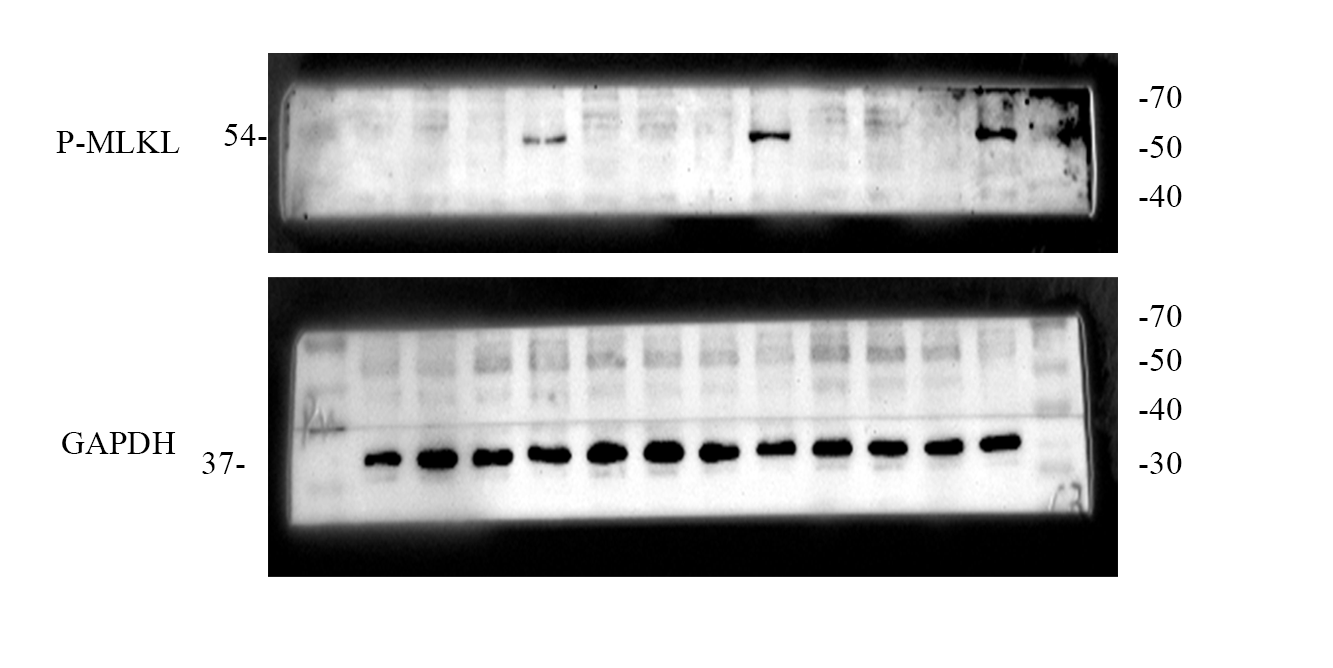


**Figure S12**: Uncropped blot images corresponding to Figure 8A P-MLKL.The same membrane was cut into strips according to the molecular weight markers to allow for simultaneous incubation with antibodies against P-MLKL and GAPDH.Lane assignments: Samples from Control (Con), Low-dose TMAO (L-TMAO), Medium-dose TMAO (M-TMAO), and High-dose TMAO (H-TMAO) groups were loaded in an alternating manner for three biological replicates.


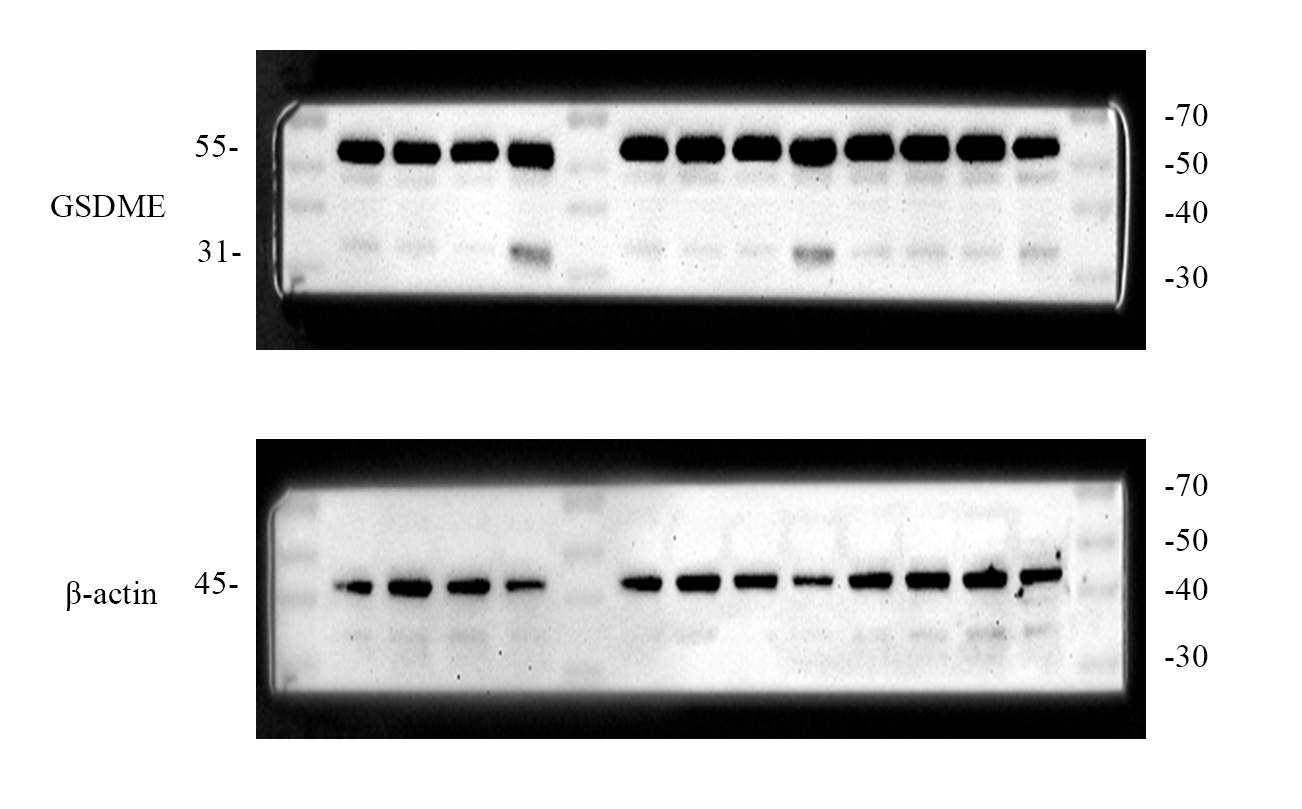


**Figure S13**: Uncropped blot images corresponding to Figure 8B GSDME.Lane assignments: Samples from Control (Con), Low-dose TMAO (L-TMAO), Medium-dose TMAO (M-TMAO), and High-dose TMAO (H-TMAO) groups were loaded in an alternating manner for three biological replicates.


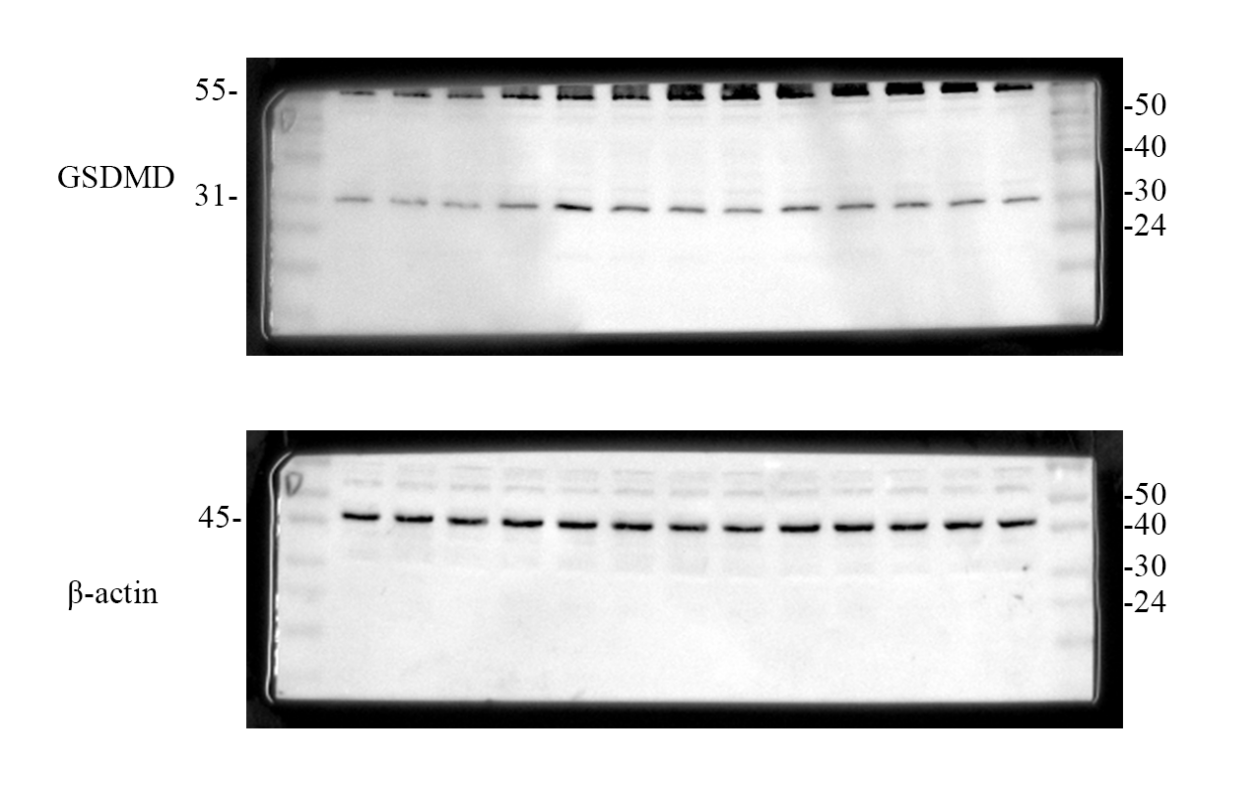


**Figure S14**: Uncropped blot images corresponding to Figure 8B GSDMD.Lane assignments: Three biological replicates for each group were loaded consecutively. Lanes 1-3: Control (Con); Lanes 4-6: Low-dose TMAO (L-TMAO); Lanes 7-9: Medium-dose TMAO (M-TMAO); Lanes 10-13: High-dose TMAO (H-TMAO).


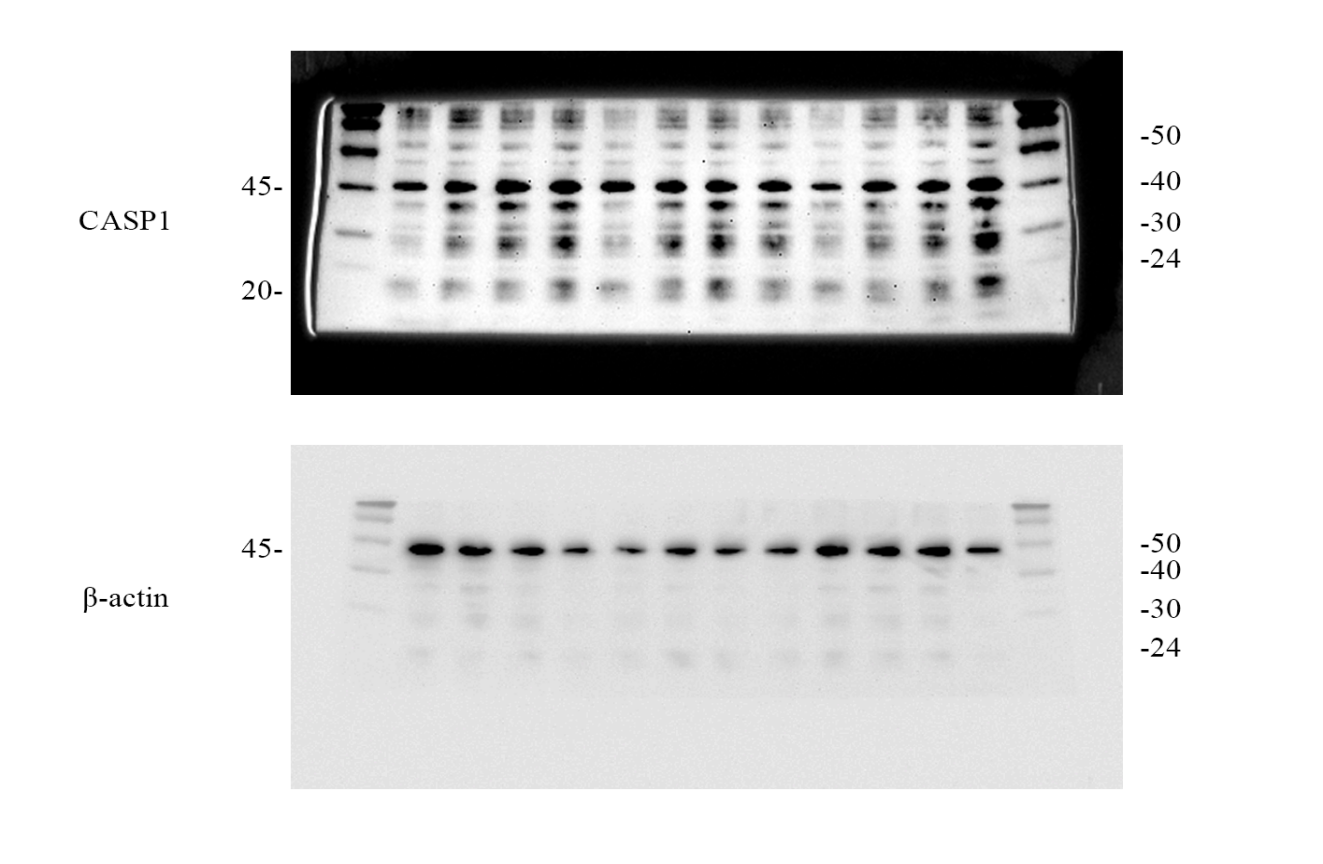


**Figure S15**: Uncropped blot images corresponding to Figure 8B CASP1.Lane assignments: Samples from Control (Con), Low-dose TMAO (L-TMAO), Medium-dose TMAO (M-TMAO), and High-dose TMAO (H-TMAO) groups were loaded in an alternating manner for three biological replicates.


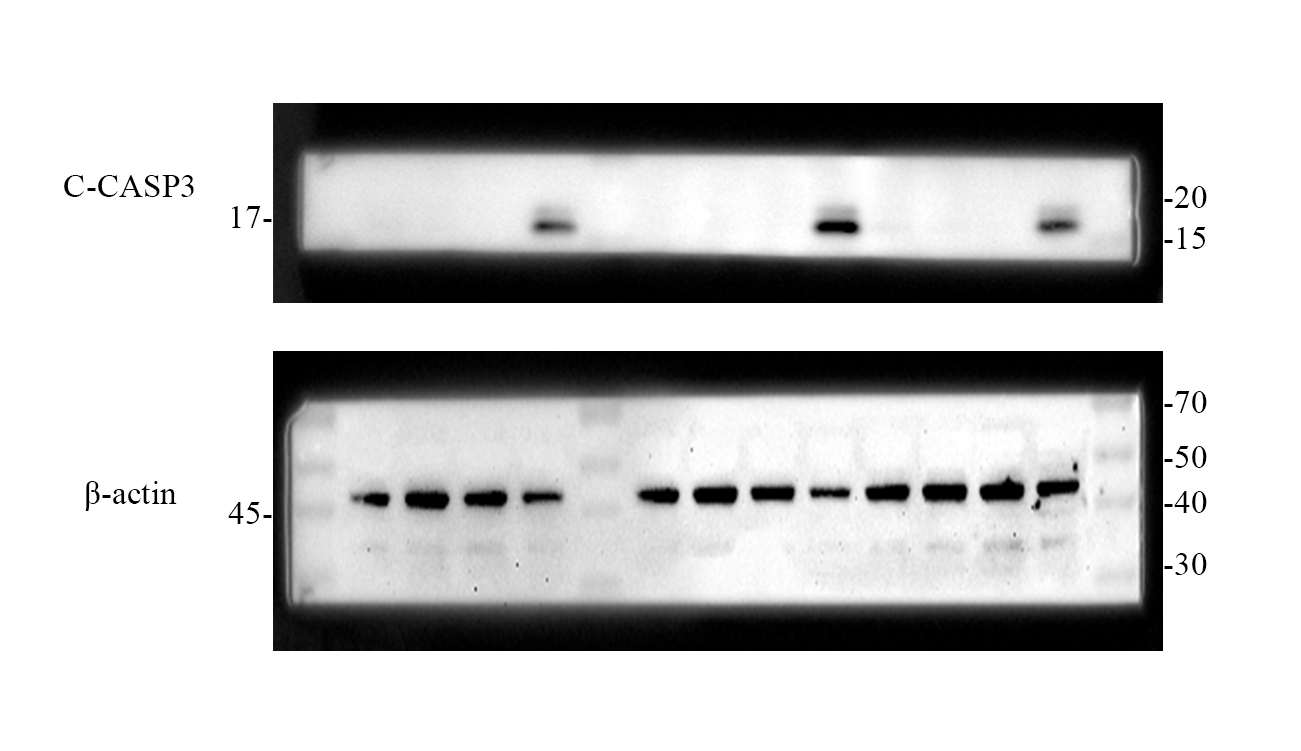


**Figure S16**: Uncropped blot images corresponding to Figure 8C C-CASP3.The same membrane was cut into strips according to the molecular weight markers to allow for simultaneous incubation with antibodies against C-CASP3, GSDME, and β-Actin.Lane assignments: Samples from Control (Con), Low-dose TMAO (L-TMAO), Medium-dose TMAO (M-TMAO), and High-dose TMAO (H-TMAO) groups were loaded in an alternating manner for three biological replicates.


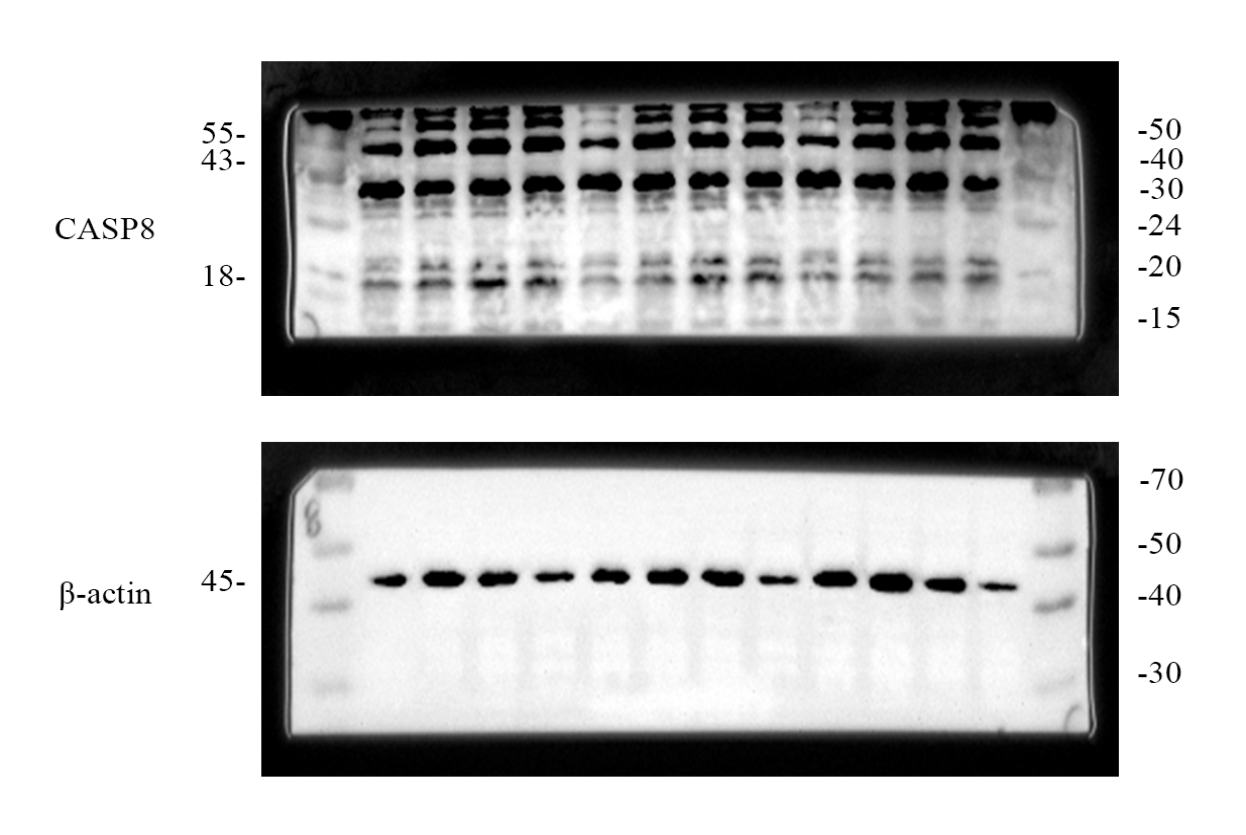


**Figure S17**: Uncropped blot images corresponding to Figure 8C CASP8.Lane assignments: Samples from Control (Con), Low-dose TMAO (L-TMAO), Medium-dose TMAO (M-TMAO), and High-dose TMAO (H-TMAO) groups were loaded in an alternating manner for three biological replicates.


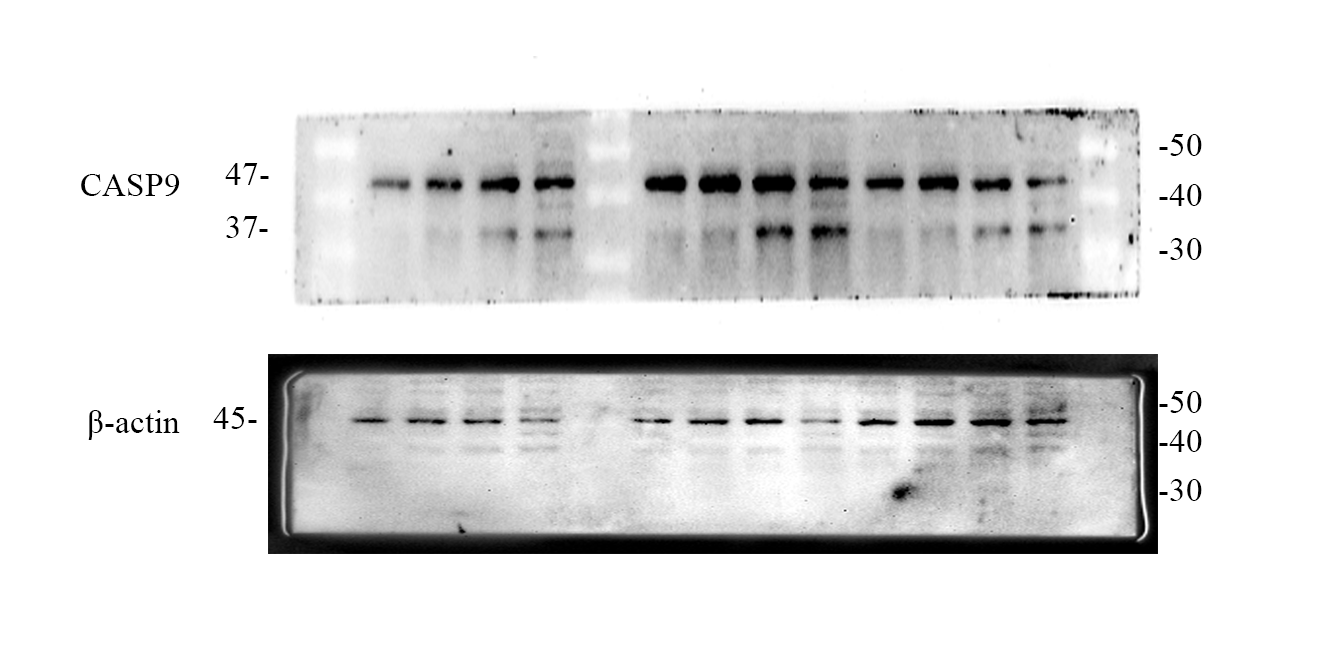


**Figure S18**: Uncropped blot images corresponding to Figure 8C CASP9.Lane assignments: Samples from Control (Con), Low-dose TMAO (L-TMAO), Medium-dose TMAO (M-TMAO), and High-dose TMAO (H-TMAO) groups were loaded in an alternating manner for three biological replicates.


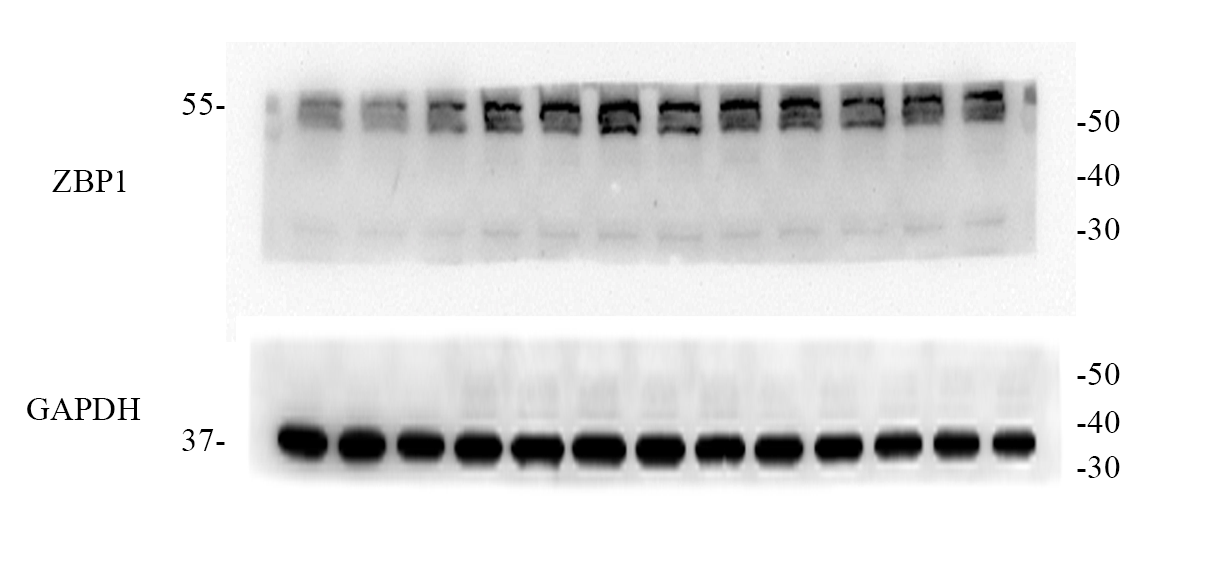


**Figure S19**: Uncropped blot images corresponding to Figure 8D ZBP1.Lane assignments: Three biological replicates for each group were loaded consecutively. Lanes 1-3: Control (Con); Lanes 4-6: Low-dose TMAO (L-TMAO); Lanes 7-9: Medium-dose TMAO (M-TMAO); Lanes 10-12: High-dose TMAO (H-TMAO).
